# Protein changes as robust signatures of fish chronic stress: a proteomics approach to fish welfare research

**DOI:** 10.1186/s12864-020-6728-4

**Published:** 2020-04-19

**Authors:** Cláudia Raposo de Magalhães, Denise Schrama, Ana Paula Farinha, Dominique Revets, Annette Kuehn, Sébastien Planchon, Pedro Miguel Rodrigues, Marco Cerqueira

**Affiliations:** 1 Centre of Marine Sciences, CCMAR, Universidade do Algarve, Campus de Gambelas, Edifício 7, 8005-139 Faro, Portugal; 2 Luxembourg Institute of Health, Department of Infection and Immunity, 29, rue Henri Koch, L-4354 Esch-sur-Alzette, Luxembourg; 3 Luxembourg Institute of Science and Technology, Environmental Research and Innovation (ERIN) Department, 5, avenue des Hauts-Fourneaux, L-4362 Esch-sur-Alzette, Luxembourg

**Keywords:** Aquaculture, Biomarkers, Cortisol, Glucose, Proteome, Mass-spectrometry

## Abstract

**Background:**

Aquaculture is a fast-growing industry and therefore welfare and environmental impact have become of utmost importance. Preventing stress associated to common aquaculture practices and optimizing the fish stress response by quantification of the stress level, are important steps towards the improvement of welfare standards. Stress is characterized by a cascade of physiological responses that, in-turn, induce further changes at the whole-animal level. These can either increase fitness or impair welfare. Nevertheless, monitorization of this dynamic process has, up until now, relied on indicators that are only a snapshot of the stress level experienced. Promising technological tools, such as proteomics, allow an unbiased approach for the discovery of potential biomarkers for stress monitoring. Within this scope, using Gilthead seabream (*Sparus aurata*) as a model, three chronic stress conditions, namely overcrowding, handling and hypoxia, were employed to evaluate the potential of the fish protein-based adaptations as reliable signatures of chronic stress, in contrast with the commonly used hormonal and metabolic indicators.

**Results:**

A broad spectrum of biological variation regarding cortisol and glucose levels was observed, the values of which rose higher in net-handled fish. In this sense, a potential pattern of stressor-specificity was clear, as the level of response varied markedly between a persistent (crowding) and a repetitive stressor (handling). Gel-based proteomics analysis of the plasma proteome also revealed that net-handled fish had the highest number of differential proteins, compared to the other trials. Mass spectrometric analysis, followed by gene ontology enrichment and protein-protein interaction analyses, characterized those as humoral components of the innate immune system and key elements of the response to stimulus.

**Conclusions:**

Overall, this study represents the first screening of more reliable signatures of physiological adaptation to chronic stress in fish, allowing the future development of novel biomarker models to monitor fish welfare.

## Background

Managing welfare of fish in captivity is of increasing importance, both for productivity and sustainability reasons [1]. There is still no clear consensus on how welfare should be defined or objectively measured, given the complexity and controversy of the concept [2, 3]. Challenges like divergent coping mechanisms, the incomplete knowledge regarding the nociceptive system of fish (e.g. emotional-like states; cognitive abilities, pain, suffering) [4–7] and the lack of reliable physiological indicators of fish welfare, make its investigation even more difficult [8].

An aquaculture rearing facility deals with multiple stressful situations (stressors) that are inherent to daily routines and can compromise the fish well-being. These situations are usually unpredictable and uncontrollable for the animal and can range in duration and severity [8, 9]. Fish launch a physiological response when faced with these threatening situations [9, 10]. This adaptive mechanism, known as stress response, involves a cascade of reactions and enables the fish to cope with the stressor. However, when a stressful event is repeated or prolonged, it exceeds the organism’s natural regulatory capacity and the fish fails to regain homeostasis, consequently impairing welfare [11, 12].

The physiological stress response starts with the immediate activation of the sympathetic response, followed by a slightly delayed activation of the hypothalamo-pituitary-interrenal (HPI) axis. As a result, catecholamines and corticosteroids (cortisol in teleosts), respectively, are released into the bloodstream [13, 14]. These hormones lead to a series of downstream responses involving alterations in the energy metabolism and respiratory and immune functions [15]. The rapid mobilization of energy substrates such as glucose (the fuel needed for the coping mechanisms) is caused by the activation of both the glycogenolysis in the liver or muscle, and the hepatic gluconeogenesis, by the catecholamines and cortisol, respectively [16, 17]. Stressful stimuli can also lead to strenuous exercise fuelled by anaerobic glycolysis in the muscle, generating lactate, which is then released into plasma [18, 19]. Prolonged exposure to the stressor will inevitably lead to alterations that are reflected in the whole-animal’s performance, like perturbations at the reproduction, immunological, growth and behaviour levels [20].

The plasma levels of cortisol, alongside glucose and lactate, are the most commonly used physiological indicators to assess stress in fish [21]. Nevertheless, some inconsistencies have been reported in several experimental studies. This demonstrates the unreliability of these indicators, mainly in cases of chronic stress, which is mostly due to: (i) a high variability of response levels; (ii) a decrease of the cortisol levels to basal levels within minutes/hours following an acute stressor; (iii) the fact that fish can adapt, to certain extent, to chronic stress and the cortisol response is therefore attenuated; and (iv) the intrinsic and extrinsic factors (e.g. age, sexual maturity, social status, level of domestication, prior experience, nutritional status) that can affect cortisol secretion [22–27]. In this sense, it is vital to complement the existing behavioural, biochemical and physiological measures for a correct interpretation of the welfare status of the fish. This will be crucial to form a robust welfare assessment and to allow, in the future, the development of targeted recommendations and legislation. With the increasing research into the welfare of cultured fish, more advanced technologies are gaining popularity. Proteomics are promising alternatives for the discovery of candidate molecular markers that can indicate physiological alterations due to stress exposure [28]. Despite the limitations to the use of these technologies in the aquaculture field [29], several studies prove already the huge potential of proteomics for the identification of stress signatures [30–34].

There is very little data available concerning the process of long-term coping with a chronic stressor and indicators used in this case. Considering this gap in research, we aim, in the present study, to comparatively assess the stress responses of fish at different levels (i.e., plasma stress markers, changes in plasma proteins’ abundance and muscle biochemistry). Using Gilthead seabream (*Sparus aurata*) as model, three chronic stress conditions were employed, and proteomics was used to benchmark potential signatures of stress adaptation in the plasma proteome since several proteins resulting from physiological events are released into circulation. Gilthead seabream was the chosen species in this study since it is one of the most important species in European aquaculture with high commercial value. This work aims to pioneer a better understanding of the underlying molecular mechanisms behind the fish physiological adaptation to long-term stress. Additionally, it aims to bridge the gap between the scientific community and the industry by paving the way for the development of novel biomarkers to monitor fish welfare.

## Results

### Fish general condition

Fish were monitored every day during the trials. The experimental periods reached the end with a 100% survival rate. The overall condition and growth performance of the fish were also monitored (Additional file 1), and initial (IBW) and final body weights (FBW) were recorded for each experiment. The average body weight was reduced by the end of the net handling (NET) and hypoxia (HYP) trials, in all groups, including the control. However, there were no significant differences in final body weights between the control group and any of the stressed groups (*P* > 0.05), suggesting that weight reductions were unrelated to the stressor.

### Plasma stress markers analysis

**Fig. 1:**
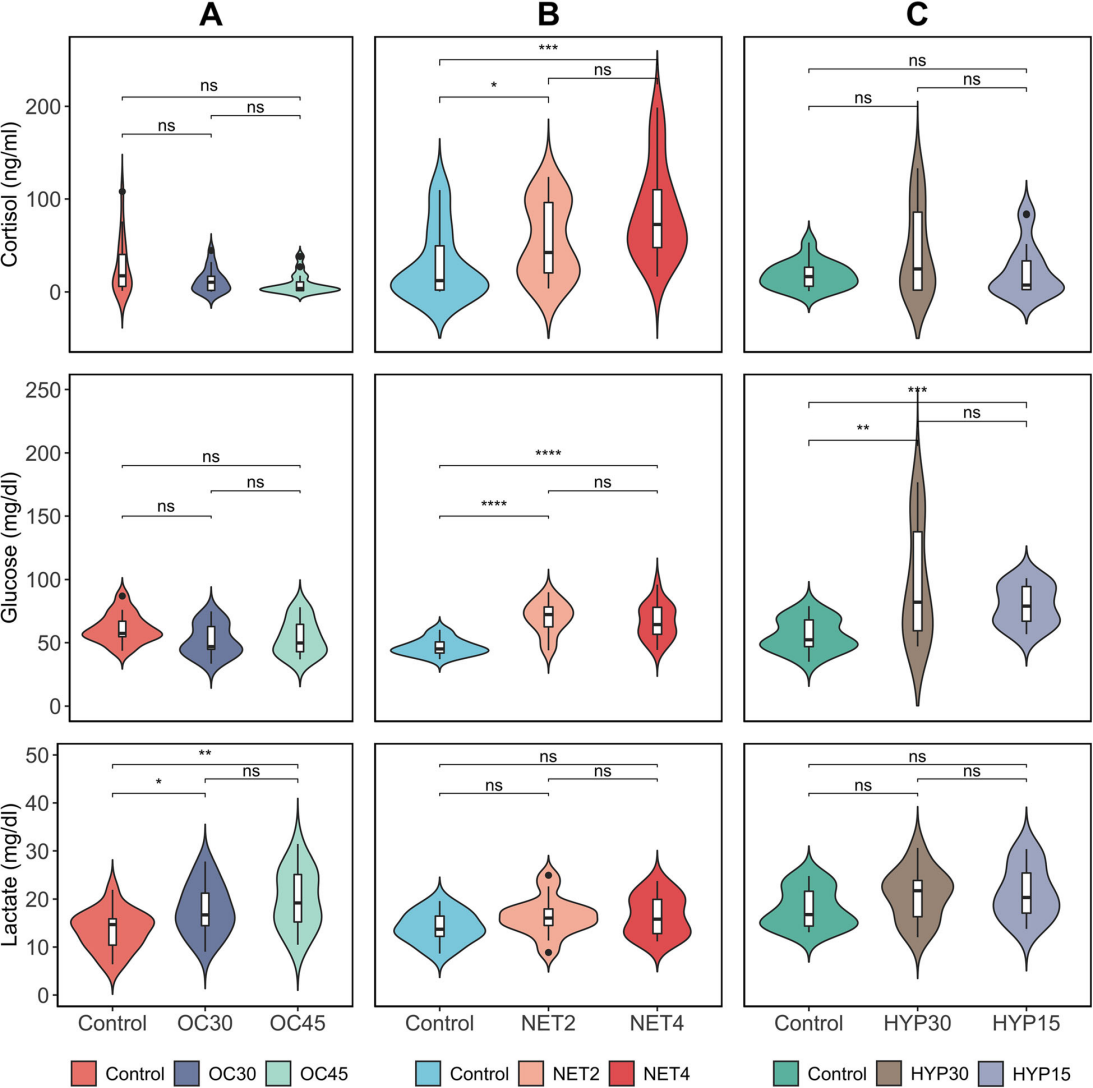
Violin plots showing the distributions of plasma cortisol (ng/ml), glucose (mg/dl) and lactate (mg/dl) levels of gilthead seabream (*Sparus aurata*) submitted to different chronic stressors (**a** – overcrowding, **b** – net handling, **c** – hypoxia), in two intensities, and unstressed fish (control) (*n* = 18). The box-plot inside includes observations from the 25th to the 75th percentiles as determined by R software; the horizontal line indicates the median value. Whiskers extend 1.5 times the interquartile range. Single data points are outlying data. *P < 0.05; **P < 0.01; ***P < 0.001; **** *P* < 0.0001. NS (not significant) indicates a *P-*value greater than 0.05

Circulating cortisol, glucose, and lactate levels were measured in Gilthead seabream submitted to different chronic stressors and in control fish (Fig.1). The overall levels of these metabolites showed a high variability of biological responses in all trials, with several data points considered outliers (outside of the interquartile range). Cortisol levels presented the highest intervals of values. In the OC trial, only lactate plasma levels presented statistically significant differences, both between control and stressed groups (Lactate_CTRL_ – 13.46 ± 4.07, Lactate_OC30–_17.79 ± 5.29, Lactate_OC45_–19.89 ± 6.19, *P* = 2.89e^− 3^). Curiously, in the case of cortisol, although not significant, stressed fish showed decreased levels compared to control (Cortisol_CTRL_ – 28.03 ± 30.78, Cortisol_OC30_–12.51 ± 13.13, Cortisol_OC45_–8.54 ± 10.92, *P* = 1.20e^− 1^). In the NET trial, statistically significant differences were registered for the cortisol and glucose plasma levels, again between control and the stressed groups (Cortisol_CTRL_ – 29.38 ± 38.06, Cortisol_NET4_–55.69 ± 41.05, Cortisol_NET4_–84.83 ± 50.77, *P* = 5.15e^− 4^; Glucose_CTRL_ – 46.57 ± 6.58, Glucose_NET2–_69.76 ± 12.90, Glucose_NET4_–66.60 ± 13.74, *P* = 2.06e^− 6^). In the HYP trial, significant differences were only observed in the glucose levels (Glucose_CTRL_ – 55.85 ± 12.72, Glucose_HYP15_–96.22 ± 45.53, Glucose_HYP15_–79.30 ± 15.78, *P* = 1.07e^− 4^). Cortisol values are presented as mean ± standard deviation (S.D.) in ng/ml, and glucose and lactate values in mg/dl.

### Post-mortem muscle biochemical changes

**Fig. 2:**
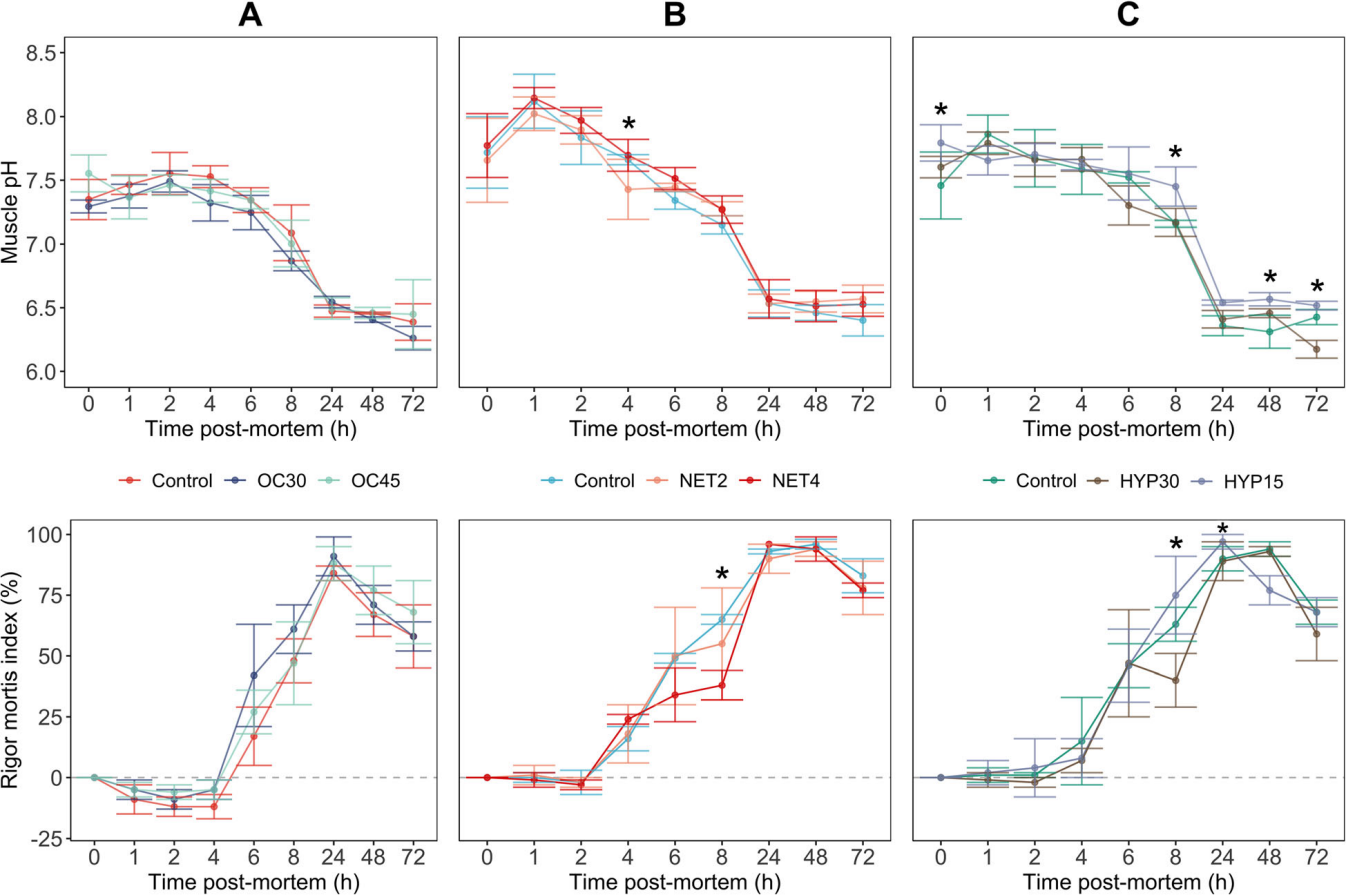
*Post-mortem* changes in muscle pH and *rigor mortis* of gilthead seabream (*Sparus aurata*) submitted to different chronic stressors (**a** – overcrowding, **b** – net handling, **c** – hypoxia), in two intensities, and unstressed fish (control), stored in ice for 72 h. Data points are the mean ± S.D. of *n* = 9 for each sampling time. Means labelled * are different at *P* < 0.05

Muscle pH declined over 72 HAD*,* in Gilthead seabream stored in ice. Values ranged from an average of 7.4, 7.7 and 7.4 immediately after slaughtering, to 6.3, 6.5 and 6.4 at the last sampling time, in fish from OC, NET and HYP trials, respectively. Significant differences between conditions were found for the NET trial at 4 (*P*_NET2-NET4_ = 0.032) and 72 HAD (*P*_CTRL-NET2_ = 0.008), and for the HYP trial at 0 (*P*_CTRL-HYP15_ = 0.021), 8 (*P*_CTRL-HYP15_ = 0.003, also in HYP30-HYP15 with lower significance), 48 (*P*_CTRL-HYP15_ = 0.006) and 72 HAD (*P*_HYP30-HYP15_ < 0.001) (Fig. 2).

The onset and resolution of *rigor mortis* (Fig.2) showed significant differences between treatments in the NET and HYP trials, specifically at 8 HAD (*P*_CTRL-NET4_ < 0.001), and at 8 (*P*_HYP30-HYP15_ < 0.001) and 24 HAD (*P*_HYP30-HYP15_ = 0.020), respectively. In the OC trial, fish reached an average maximum rigor strength at 24 HAD. In the NET trial, averaged maximum rigor strength was reached at 48 HAD in CTRL and NET2, and at 24 HAD in NET4 group. In the HYP trial, all groups reached averaged maximum rigor strength at 48 HAD.

### Plasma proteomics analysis

**Fig. 3:**
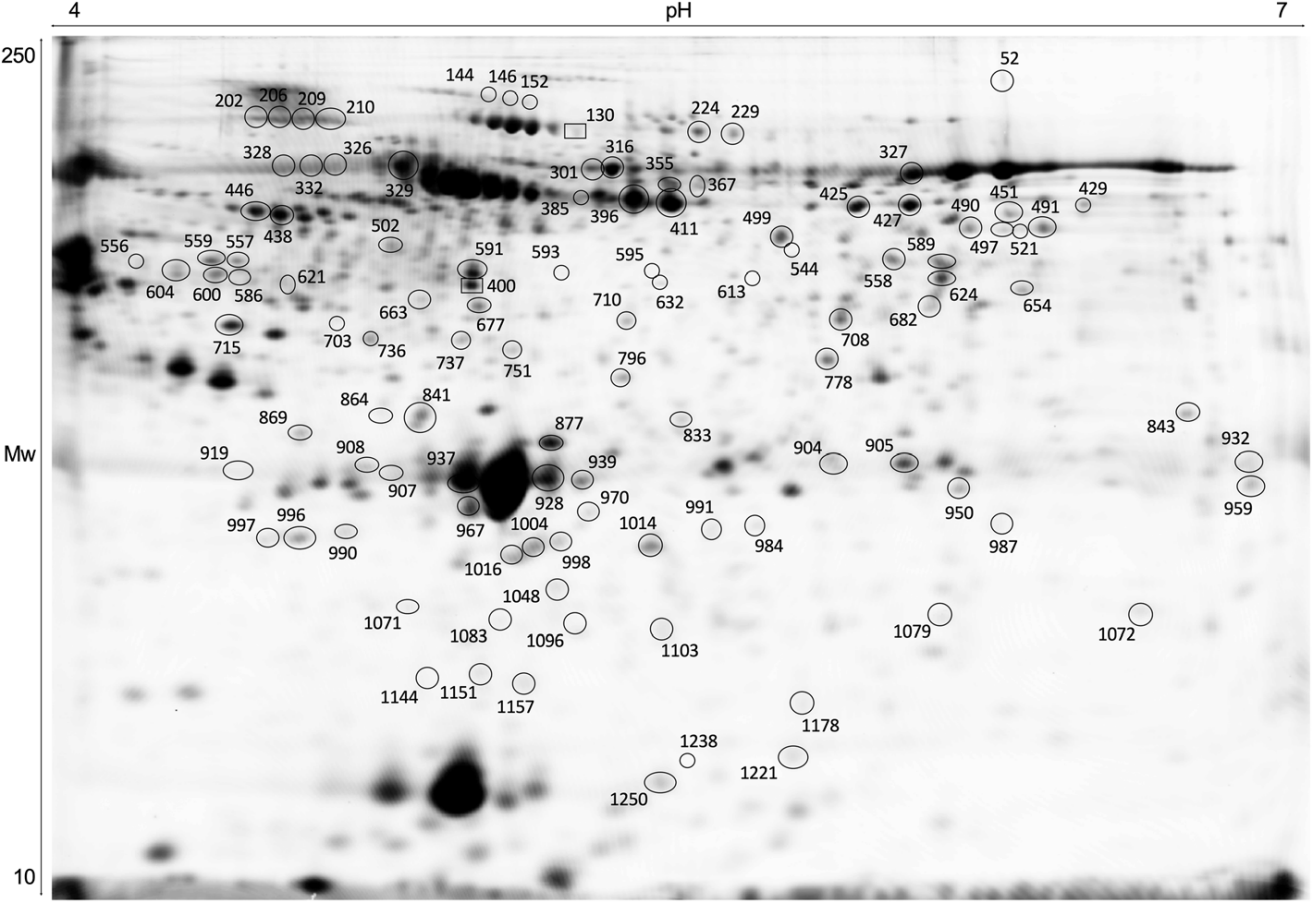
Representative pattern of gilthead seabream (*Sparus aurata*) blood plasma on a 12.5% polyacrylamide 2D gel. Black circles represent the 107 proteins identified by MALDI-TOF/TOF MS with significant differences in abundance in NET groups and black squares the 2 proteins with significant differences in abundance in HYP groups (*P* < 0.05)

A comparative proteomics analysis of the Gilthead seabream plasma between the control and the stress treatments detected, 681, 752 and 681 protein spots for the OC, NET and HYP trials, respectively, within the pH range of 4–7 and a molecular mass range of 11–114 kDa. After statistical analysis, 19, 360 and 34 protein spots within the OC, NET and HYP trials, respectively, were found to present significantly differential abundance (significance threshold at *P* < 0.05) between experimental conditions. From these, 7, 171 and 12 were manually excised from the 2D gels for MALDI-TOF/TOF MS analysis. No proteins were identified with significance for the OC trial. For the NET and HYP trials, 107 and 2 differential protein spots, respectively, were successfully identified by a combination of PMF and MS/MS search, with significant scores (protein score > 76, total ion score > 60, *P* < 0.05). Among the spots identified from the NET trial, 13 showed more than one significant protein identification (202, 326, 521, 559, 586, 604, 677, 877, 950, 959, 990, 996 and 1157), indicating that multiple proteins migrated to the same spots on the gel. The identified proteins are listed in an additional file (see additional file 2). A representative 2D-gel of the Gilthead seabream plasma proteome is shown in Fig. 3.

**Fig.4:**
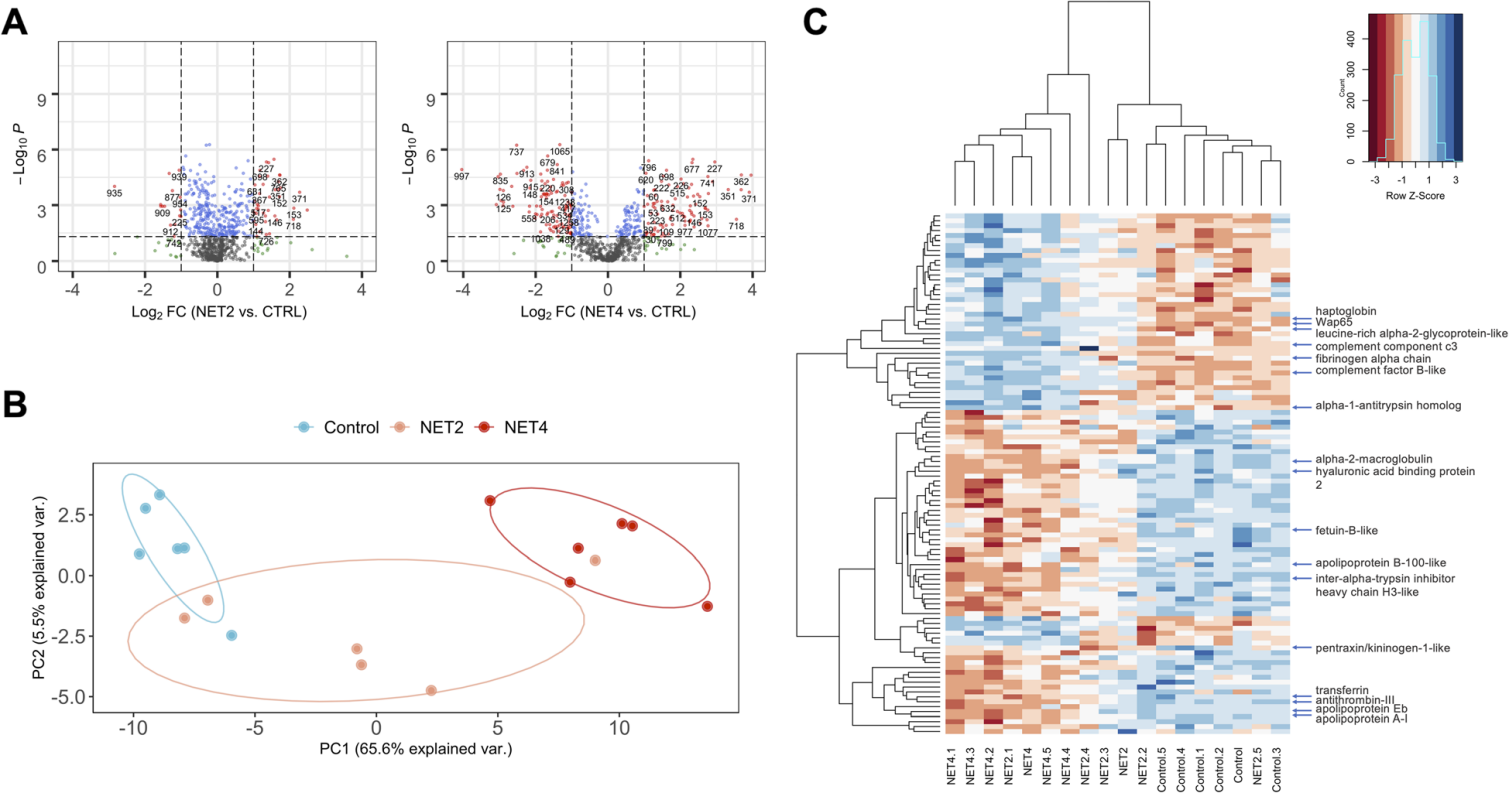
**a** – Volcano plots of the entire set of plasma proteins detected by DIGE analysis on the NET trial samples. Each point represents the difference in abundance (fold-change) between stressed fish (NET2 on the left; NET4 on the right) and control fish plotted against the level of statistical significance. Dotted vertical lines represent a 2-fold variation in abundance, while dotted horizontal line represent the significance level of *P* < 0.05. Red dots represent proteins significantly up- and down-regulated. **b** – Principal component analysis performed with the normalized spot volumes of the 107 identified proteins in the plasma samples of gilthead seabream from the NET trial (*n* = 6). Blue, orange and red dots represent CTRL, NET2 and NET4 groups, respectively. **c** – Hierarchical clustering of 107 significantly differential proteins identified in the plasma samples of gilthead seabream from net handling (NET) trial. Rows represent expression patterns of individual proteins, while each column corresponds to a biological replicate (fish). Cell colour indicates the normalized Z-scores of the spot volumes

Considering the number of identifications in each trial, only the 107 identified protein spots from the NET trial were considered for further statistical and bioinformatics analyses. At this step, a log-fold change cut-off of ±1.0 (*P* < 0.05) was applied (Fig. 4-a) and a total of 56 identified protein spots (corresponding to 20 single entries) were considered significant. From these, 19 were up-regulated in stressed fish and 34 were down-regulated. Three spots (502, 990 and 1021) showed multi-expression patterns and could not be classified as up- or down-regulated. Seventeen protein spots (502, 715, 841, 905, 908, 919, 937, 939, 967, 997, 1004, 1016, 1021, 1151, 1221, 1238 and 1250) were identified as apolipoprotein A-I, whereas 13 were down-regulated in stressed fish. Four spots (864, 869, 990 and 996) were identified as apolipoprotein Eb and 2 were up-regulated. Complement factor B was identified in 4 spots (144, 146, 152 and 737) and complement component C3 in 5 spots (591, 593, 595, 1048 and 1083) from which 3 from each were up-regulated. Two protein spots (796 and 833) were identified as warm-temperature acclimation-related 65 kDa, 1 down- and 1 up-regulated. Three spots (202, 206 and 209), identified as inter-alpha-trypsin inhibitor heavy chain H3, 2 spots (224 and 229) as alpha-2-macroglobulin and 5 (558, 751, 843, 904 and 1079) identified as transferrin were down-regulated. Two spots (663 and 710), identified as haptoglobin, were found to be up-regulated. Fibrinogen alpha-chain was identified in two spots (521 and 544) and were both up-regulated. Alpha-1-antitrypsin homolog, apolipoprotein B-100, beta-actin, calcium/calmodulin-dependent protein kinase type II, leucine-rich alpha-2-glycoprotein, fetuin-B-like, hemopexin-like, hyaluronic acid-binding protein 2 and pentraxin were identified in a single protein spot each.

Hierarchical clustering (HCA) and principal component (PCA) analyses were performed for the identified 107 proteins spots with differential relative abundance across NET groups to check how well the samples grouped based on the expression patterns of the protein spots. The PCA (Fig.4-b) showed two main clusters belonging to the control and NET4 samples, while 2 biological samples belonging to the NET2 group clustered together with the control samples and 1 with the NET4 samples. The 107 differential protein spots were centralized into two principal components (PC), PC1 and PC2, which represented the maximum variation (65.6%) and the next highest variation (5.5%), respectively. The HCA (Fig. 4-c) likewise revealed two main groups regarding the biological replicates, as observed by the top dendrogram. The protein spots were also grouped into two main clusters, one displaying a pattern of higher relative abundance and the other of lower relative abundance in stressed fish, when compared to the control. As described above for the PCA, higher variability in NET2 was also shown in the HCA.

**Fig.5:**
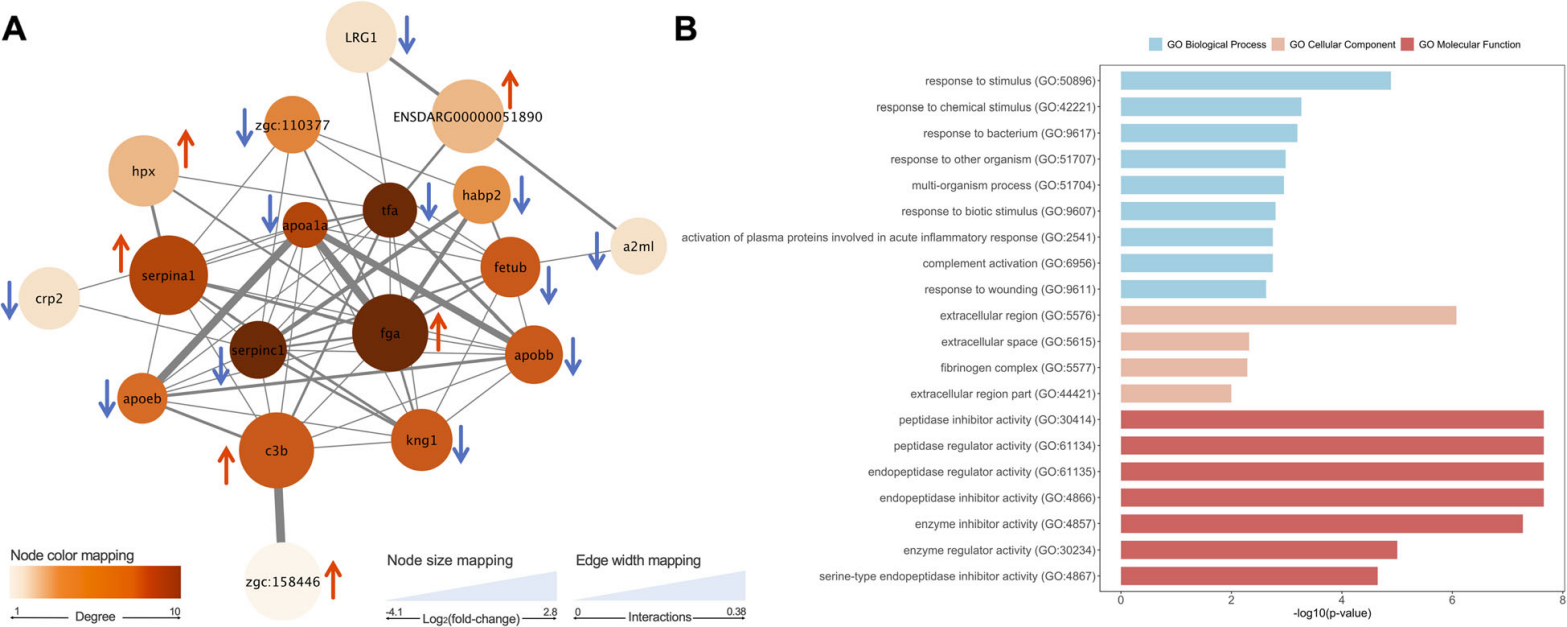
**A** – Protein-protein interaction network generated with 18 differential proteins identified in the plasma of fish from NET trial. Nodes represent proteins and edges the functional associations between them. STRING annotations are described in Table 1. Red arrows represent up-regulated proteins in both treatments; blue arrows represent down-regulated proteins in both treatments. D – GO Enrichment analysis of the 18 proteins showing significantly differential abundance between control and NET treatments (hypergeometric test, FDR < 0.05)

For the network and GO enrichment analyses the subset of 20 single protein identifications mentioned above was blasted against *Danio rerio* in the UniprotKB database. A PPI network (Fig.5-a) was generated on the STRING web tool revealing 61 edges among 18 nodes/proteins (2 proteins had no interaction with the main network), with a clustering coefficient of 0.677 and a very significant enrichment value (*P* < 1.0e^− 16^). The analysis was performed on Cytoscape and specific topological parameters were selected to demonstrate the importance and distribution of the nodes in the network: a darker colour intensity of the nodes indicates higher degree, while the size was estimated using the variation in protein abundance (fold-change). For every single entry, one protein spot was chosen as the most representative of each protein (Table 1), based mainly on the protein score and experimental molecular weight and pI close to the theoretical ones. From these 18 spots, 11 were down- and 7 were up-regulated, however, these differences in abundance were mostly significant (log-fold change > 1.0 or < − 1.0, *q-value* < 0.05) for the NET4 treatment (only 2 were exclusively significant for the NET2 treatment and 2 were significant for both treatments). Thus, the fold-change of these 18 spots between NET4 and CTRL groups was used to estimate the size of the nodes on the PPI network, which ranged from − 4.04 to + 2.78. SERPINC1 (antithrombin-III), TFA (transferrin) and FGA (fibrinogen alpha-chain) occupied the most central positions in the network having the highest number of interactions, while APOA1 (apolipoprotein A-I) showed the highest number of experimentally demonstrated interactions, mainly with APOEB (apolipoprotein Eb), APOBB (apolipoprotein B-100) and FGA. GO Enrichment analysis (Fig. 5-b) revealed 19 overrepresented (hypergeometric test, FDR < 0.05) GO Biological Process (BP) terms, mostly linked to the immune system and response to stimulus. No annotations were retrieved for alpha-1-antitrypsin, leucine-rich alpha-2-glycoprotein-like, apolipoprotein A-I, apolipoprotein B-100-like, haptoglobin, pentraxin and hyaluronic acid-binding protein 2. In the horizontal bar plot (Fig. 5-b), only the 9 most significant terms are represented. GO Molecular function enrichment analysis accounted for 8 terms with 5 main proteins (alpha-1-antitrypsin, antithrombin-III, inter-alpha-trypsin-inhibitor, kininogen and alpha-2-macroglobulin) while GO Cellular component revealed 4 enriched terms with 2 main proteins (fibrinogen alpha-chain and alpha-2-macroglobulin). A complete list of all GO terms is described on the additional file 3.

**Table 1:** String annotations and fold-changes of the proteins in the PPI network. Bold lettering in the “FC” column indicates significant fold-changes (> 1.0 and < − 1.0). List is given in ascending order of spot number

Spot^a^	Accession no.^b^	Protein ID^c^	FC^d^		***Danio rerio*** homolog (UniprotKB identifier)	String annotation
			NET2	NET4		
152	XP_008277007.1	PREDICTED: complement factor B-like [*Stegastes partitus*]	**1.72**	**2.54**	F1QFT0	zgc:158446
202	XP_010753395.2	PREDICTED: antithrombin-III [*Larimichthys crocea*]	−0.36	**−1.61**	Q8AYE3	serpinc1
209	XP_019111370.1	PREDICTED: inter-alpha-trypsin inhibitor heavy chain H3-like [Larimichthys crocea]	−0.49	**−1.76**	F1QTF9	zgc:110377
224	XP_017260893.1	alpha-2-macroglobulin, partial [Kryptolebias marmoratus]	−0.62	**−1.74**	A0A0R4IDD1	a2ml
316	AWP20152.1	putative apolipoprotein B-100-like isoform 2 [*Scophthalmus maximus*]	−0.88	**−1.46**	Q5TZ29	apobb
367	XP_023285742.1	alpha-1-antitrypsin homolog [*Seriola lalandi* dorsalis]	1.17	**2.78**	Q6P5I9	serpina1
544	KKF22678.1	Fibrinogen alpha chain [Larimichthys crocea]	1.33	**2.26**	B8A5L6	fga
556	XP_018550494.1	PREDICTED: leucine-rich alpha-2-glycoprotein-like [*Lates calcarifer*]	0.51	**1.20**	Q5RHE5	LRG1
558	AEA41139.1	transferrin [*Sparus aurata*]	−0.46	**−2.18**	A0A2R8RRA6	tfa
595	ADM13620.1	complement component c3 [Sparus aurata]	1.08	**2.06**	Q3MU74	c3b
710	ARI46218.1	haptoglobin [Sparus aurata]	**1.39**	**1.53**	F8W5P2	ENSDARG00000051890
736	AJW65884.1	Hyaluronic acid binding protein 2 [Sparus aurata]	−0.33	**−1.53**	Q1JQ29	habp2
796	ACN54269.1	warm temperature acclimation-related 65 kDa protein [Sparus aurata]	0.85	**1.13**	Q6PHG2	hpx
877	BAM36361.1	pentraxin [*Oplegnathus fasciatus*]	**−1.24**	−0.73	Q7SZ53	crp2
	XP_022604055.1	kininogen-1-like [*Seriola dumerili*]	**−1.24**	−0.73	Q5XJ76	kng1
996	APO15792.1	apolipoprotein Eb [Sparus aurata]	−0.10	**−3.04**	O42364	apoeb
997 1072	XP_010742296.3	apolipoprotein A-I [Larimichthys crocea]	−0.92	**−4.04**	O42363	apoa1a
	XP_020489366.1	fetuin-B-like [*Labrus bergylta*]	−0.47	**−1.07**	E7FE90	fetub

^a^ Spot no. – number of the spot in the 2D gel (Fig.3), attributed by the SameSpots software

^b^ Accession number – NCBI accession number

^c^ Protein ID – protein identification by MALDI-TOF/TOF MS

^d^ FC - Log2(fold-change) - significant changes in protein abundance (treated/control). Bold lettering indicates significant fold-changes (> 1.0 and < − 1.0)

## Discussion

In this study, the stress response of farmed Gilthead seabream adults to chronic stress conditions was primarily assessed by observing both changes in the concentration of routine plasma stress indicators, namely cortisol, glucose and lactate, and *post-mortem* biochemical parameters, explicitly pH and *rigor mortis*. To evaluate the existence of possible unbiased and reliable markers of chronic stress, proteomics was used to verify the potential of fish protein-based adaptations.

Cortisol is the most commonly used physiological indicator of the primary response to stress [21]. However, it has been shown that this corticosteroid is not a reliable biomarker of long-term stress exposure [27, 35–37]. In this study, Gilthead seabream that endured high stocking densities over 54 days showed a possible reconfiguration of the cortisol response. This is supported by the observed downward trend of this metabolite, as compared to unstressed fish. This is suggestive of either a non-activated or an altered responsiveness of the HPI axis, which sometimes leads to the hyporeactivity of the corticosteroid response [38]. The same outcome was observed in juvenile Gilthead seabream confined for 14 days at 26 kg/m^3^ [39] and in meagre, cultured at different stocking densities for 40 days [40]. In the NET trial, however, apart from the wide dispersion of observations, plasma cortisol levels were significantly higher in handled fish. This result suggests that the fish were not able to appropriately adapt to the handling stressor. Its persistence, unpredictability and severity, could have prevented the possibility of habituation. Regarding the HYP trial, no effect of the 48 h of hypoxia was reflected in these fish. This suggests an acclimation to the low oxygen environment by a possible adjustment of the oxygen requirement (e.g. reduction of high energy behaviours).

Overall, the aforementioned observations suggest that the cortisol response and the capacity of adaptation are modulated by the nature, duration and intensity of the stressor. However, other factors like species, age, sex and individual coping mechanisms seem to be ubiquitous and impact their adaptive processes [24, 38, 41]. This process of stress habituation was already suggested and demonstrated in other studies [37, 42], but this mechanism is not yet well-understood. High individual variability was also observed in each trial, most likely due to individual differences in the stress response related to intrinsic factors of the animal (e.g. coping styles, cognitive perception) [39, 43, 44]. Additionally, values registered for control fish, in every trial, are higher than the reference values reported in the literature for this species [45]. These discrepancies may have several causes, which is why cortisol should be used with caution when evaluating the magnitude of the stress response. Moreover, the difficulty of measuring resting cortisol levels is also acknowledged to be one of the causes of these discrepancies. The lack of proper planning when sampling cortisol, or the manipulation needed to net and anaesthetize the fish, can result in high “control” cortisol levels that do not correspond to the “genuine” basal levels i.e., the non-manipulated fish levels. Also, it is well-established that following the perception of an acute stressor, the levels of circulating stress markers increase within the first minutes or hours of stress response, returning to basal levels as time elapses, usually within 24 h [17, 46, 47].

Secondary physiological responses are characterized by an increase in glucose and lactate levels in blood plasma in order to satisfy the increased energy expenditure. Changes in glucose usually follow similar trends as cortisol after the stressor [14]. This is corroborated in this study by the levels of plasma glucose registered in all trials (Fig. 1). Glucose levels, besides following the same trend as cortisol levels, are, in general, below the basal values for this species [45]. This could be related to the fish’s inability to maintain the same levels of glucose in the blood due to the high demand for glucose mobilization to other tissues. The decrease of plasma glucose levels in OC is in agreement with the decrease in the cortisol levels, supporting the hypothesis of habituation or exhaustion of the endocrine system [27]. The significant increases in the plasma glucose levels of stressed fish from NET and HYP trials are consistent with previous studies. These showed that glucose rises during air exposure or low oxygen levels, due to stimulation of muscle glycogenolysis and hepatic gluconeogenesis, where glucose is synthesized to maintain the energetic substrates’ demand [48]. Similar to cortisol, glucose and lactate circulating levels also return to basal levels within hours post-stressor, which further makes of these metabolites unreliable markers in case of prolonged stressors [49, 50]. Additionally, studies also demonstrate that glucose variations in the blood are not only hormonal-induced due to stressful practices. Factors like variations in the water temperature and pH, anaesthesia, diet composition or fasting can also affect plasma glucose levels [51, 52].

When insufficient oxygen is available to maintain the aerobic ATP production, fish resort to anaerobic metabolism to meet cellular requirements. This shift consequently leads to lactate accumulation in the muscle [19, 53]. In this study, changes in the circulating lactate levels do not follow the same trends of cortisol and glucose variations. Statistically significant differences in the lactate levels were only observed in the OC trial. In this case, if the cortisol response is indeed lower due to HPI-axis acclimation, as suggested before, the lactate recycling rate in the hepatic glycogenolysis is reduced, explaining the significant plasma lactate increase in stressed fish. Additionally, previous studies show that during hypoxia or intense swimming activity, fish produce lactate in the muscle at a higher rate than it can be processed by other tissues [53].

*Post-mortem* muscle pH and *rigor mortis* have been used as tissue indicators of *ante-mortem* stress in numerous fish species [54–56]. After the fish death, both blood circulation and oxygen supply cease. The major source of ATP to the muscle is thus lost, since glycogen can no longer be oxidized. However, for a limited time after death, ATP in the muscle is maintained at a definite level by creatine kinase. Consequently, the depletion of ATP reserves stimulates the breakdown of glycogen by anaerobic glycolysis in the muscle, in order to maintain the energy expenditure. This process results in the accumulation of lactic acid, generating H^+^ ions and consequently lowering muscle pH [57]. Glycolysis continues until all glycogen is consumed or the glycolytic enzymatic system is made inactive by the low pH. Hence, the magnitude and rate of this pH fall depend on the fish’s energy reserves prior to death. These energy reserves can be influenced by the intensity and duration of the stress while fish is alive. To our knowledge, no studies were performed with Gilthead seabream regarding the effects of long-term chronic stressors on the evolution of *post-mortem* biochemical processes in the muscle. Results from this study (Fig. 2) followed the same pH trends as previous studies on gilthead seabream [58, 59], however, comparing this with the existent studies on pre-slaughter stress [55, 60, 61], muscle pH values immediately after death are lower than the ones found in this study, suggesting that stress at slaughter was low in our fish. Poli et al. 2005 state that in cases of exposure to a chronic stressor for a long time before death, the lactic acid produced can be gradually cleared from the muscle, but simultaneously the energy sources, like glycogen, will likewise gradually become exhausted. Hence, when the fish is killed, muscle pH does not suffer a dramatic fall due to an early end of *post-mortem* anaerobic glycolysis caused by energy source scarcity. This might explain the significant differences found in the HYP trial, where the highest pH values were observed in the highly stressed fish (HYP15), suggesting that these fish had lower energy reserves. Nevertheless, pH values registered after the 24 HAD, in every treatment, are in agreement with the reported by previous studies in this species at the same sampling times [58, 62].

*Rigor mortis* is inextricably correlated with muscle ATP and the pH decline. The onset of *rigor mortis* occurs with ATP depletion. When ATP reaches low levels, actin and myosin in the muscle bind together, forming the actomyosin complex and causing stiffness of the fish body [63]. A strong relationship between low muscle pH immediately after death, and a rapid onset of the rigor state was demonstrated in a range of fish species [57, 60]. In this study, the evolution of *rigor mortis* (Fig. 2) was similar between treatments and significant differences were only found in the NET and HYP trials at 8, and at 8 and 24 HAD, respectively. A delayed onset was observed, starting between 2 and 6 HAD in every trial and reaching the maximum rigor index between 24 and 48 HAD. This delay is in agreement with the high muscle pH registered immediately after death, supporting the hypothesis of low energetic reserves in our fish at the time of death. Measuring glycogen and ATP content in the fish muscle and liver would be a complementary assessment to infer about the energetic reserves and corroborate our hypothesis.

Plasma proteins were evaluated in this study since blood plasma is a very informative biological fluid, acting as a mirror of the physiological condition of the organism. Stress and stress-related hormones are recognized as modulators of the fish immune system [64], however, responses depend on the intensity and duration of the stressor. The innate immune system is a fundamental defence mechanism in fish [65]. The acute phase response is part of this system and it is mainly regulated by cytokines and glucocorticoids [66]. This response is characterized by the release of acute-phase proteins (APP), by the hepatocytes, into circulation [67]. APP can be classified as “positive” or “negative” depending on whether their plasma concentration increases or decreases during activation of this response [68]. The response profile of our fish demonstrated the same tendency of protein changes.

In this study, the pattern of protein changes observed in the plasma indicate that the fish’s immune system was affected mainly by net handling and hypoxia stressors. Nevertheless, net handling was shown to be the most impacting. The levels of 20 different plasma proteins (distributed by 56 significantly differential spots), all related with immunological processes, were shown to be modulated by repetitive net handling, compared to two proteins modulated by hypoxia. As mentioned previously, the same proteins were often detected from different spots on the 2D gels. Such a phenomenon may be due to existent isoforms or caused by adaptive changes of the proteome in an attempt to maintain cellular homeostasis. This adaptation may involve changes at the level of protein degradation, localization, function and activity – all of which can be modulated by post-translational modifications (PTMs) [69]. PTMs can regulate fundamental biochemical processes and be more energetically efficient than altering protein abundance, constituting potential interesting signatures of stress. Studies on PTMs in fish are still scarce.

The changes detected in protein abundance (listed in additional file 2), along with the PPI network and GO enrichment analyses (Fig. 5) performed, confirmed the involvement of several components of the innate immune system in the physiological adaptation to these stressors. Proteins considered to be “positive” APP were likewise shown to be increased in abundance in the plasma of fish stressed by net handling (fibrinogen alpha-chain, complement component C3, haptoglobin, complement factor B, warm-temperature acclimation 65 kDa protein, alpha-1-antitrypsin), while proteins considered as “negative” were decreased (transferrin, inter-alpha-trypsin inhibitor, apolipoprotein A-I) [70]. A diverse number of proteins involved in the APR was also found previously to be modulated in chronically stressed gilthead seabream [71].

Apolipoprotein A-I (Apo-AI) was modulated only by net handling stress. Seventeen proteoforms were identified in the plasma proteome map, being mostly decreased in abundance when comparing with the unstressed fish. Apo-AI is the main protein constituent of the high-density lipoprotein (HDL), playing a role in lipid metabolism and participating in the reverse transport of cholesterol from tissues to the liver [72, 73]. Apo-AI was also found to be decreased in abundance in crowded Atlantic salmon [74]. In cod (*Gadus morhua*) it acted as a negative regulator of the complement system [75]. Other two apolipoproteins were also found to be down-regulated in the plasma of fish from NET2 and NET4 groups (Apolipoprotein Eb and apolipoprotein B-100).

The complement system is an essential part of the innate immune system which can be activated through three pathways: the classical, alternative and lectin pathways [76]. Fish display a plethora of complement components, mainly complement component C3 (C3), which may present around five proteoforms in a single species [77]. C3 is one of the most abundant proteins in the plasma and plays a central role in the innate immune system, supporting the activation of all three pathways [76]. In this study, C3, identified in 5 proteoforms, and complement factor B (Bf), identified in 4, were found to be increased in abundance by net handling. Contrarily, C3 was down-regulated in fish exposed to low oxygen levels. Bf also plays a role in complement activation by acting as the catalytic subunit of C3 convertase, an enzyme responsible for the proteolytic cleavage of C3, in the classical and alternative pathways [76].

Several metal-binding proteins, existent in the plasma of vertebrates, can chelate iron, zinc and copper, which are essential elements for the virulence of bacteria [78]. Alpha-2-macroglobulin (A2M) is a multifunctional protein [79] found to be down-regulated in the plasma of fish submitted to handling stress. It is mostly known to act as a broad range serine proteinase inhibitor and to bind metal ions [78]. Contrarily, haptoglobin, which is also responsible for the sequestration of iron by binding to hemoglobin, was found to be increased in the plasma of handled fish. Similarly, warm-temperature acclimation-related 65 kDa protein (Wap65), which is involved in the scavenging of free heme [80], was increased in abundance by net handling and hypoxia stressors. Wap65 in fish is the homologue of mammalian hemopexin [81] and in most teleosts presents two proteoforms [82]. In this study, two spots were also matched to this protein suggesting the presence of these two proteoforms. Transferrin (Tf) decreased in abundance in the plasma of fish stressed by net handling. Tf is a plasma protein also capable of binding iron and an important constituent of the iron homeostasis [33].

In fish, antiproteases are important participants of the non-specific humoral immune defence mechanism [70]. A2M is an important factor in this mechanism. Alpha-1-antitrypsin is a serine protease inhibitor, up-regulated in net-handled fish, which is responsible for negatively regulating blood clotting molecules to prevent thrombosis [83]. Inter-alpha-trypsin inhibitor H3 is also a serine protease inhibitor, which was found to be down-regulated in the plasma of fish from NET groups. The same pattern of protein changes was verified for fetuin-B, a cysteine proteinase inhibitor recently described in teleosts [84]. Finally, fibrinogen alpha-chain, a beta-globulin involved in blood clotting, an integral part of innate immunity [83], was found to be up-regulated in the plasma of fish belonging to NET groups.

## Conclusions

In summary, the overall results suggest that physiological changes were higher in fish exposed to repeated handling, while mild and permanent stressors may allow the fish to refine their physiological processes and adapt to certain challenges. The variability in the response levels of cortisol, glucose and lactate, in fish from the same groups, alongside the possible adaptation suggested by the results, demonstrate that these indicators may not be the most robust in case of chronic stress monitoring. On the other hand, plasma proteomics allowed the detection of a cohesive network of protein changes associated with essential immunological pathways in stressed fish. These proteins will be useful in understanding the biological processes behind protein-based stress adaptation in fish and may, therefore, represent the first screening for potential biomarker candidates of chronic stress in gilthead seabream. This work is the first step for a more scientific and reliable assessment of fish welfare. A multidisciplinary approach, and the study of the stress response from the molecular to the behavioural level might just be the holistic approach needed to achieve such a goal.

## Methods

### Animals

Gilthead seabream (*Sparus aurata*) were obtained from a commercial fish farm (Maresa, Mariscos de Estero S.A., Huelva, Spain) and kept under quarantine conditions for a 2-week period at the Ramalhete Research Station (CCMAR, University of Algarve, Faro, Portugal). The fish were then individually weighed and distributed among conical fiberglass tanks (500 L), according to the density requirements of each trial. The tanks were supplied with natural flow-through seawater from Ria Formosa, and kept under natural temperature (13.4 ± 2.2 °C) and photoperiod, salinity at 34.7 ± 0.8 ‰, and artificial aeration (dissolved oxygen above 5 mg. L − 1). Fish were fed by hand once a day, with a diet manufactured by AquaSoja Portugal, following the species’ nutritional requirements.

### Experimental design

The study was performed in three separate trials: [1] Overcrowding (OC), [2] Net Handling (NET) and [3] Hypoxia (HYP), due to logistic issues. Each trial followed a 2-week acclimation period and the initial rearing density was established at 10 kg/m^3^ (except in the experimental groups of high stocking densities). In the OC trial, during the 54 days of experiment, fish (initial body weight (IBW) = 372.33 ± 6.55 g) were stressed using different high stocking densities, by increasing the number of fish in the tanks. Three different experimental groups were tested in triplicate: Control – 10 kg/m^3^ (OC_CTRL_), medium density – 30 kg/m^3^ (OC_30_), high density – 45 kg/m^3^ (OC_45_). The NET trial lasted for 45 days and the fish (IBW = 375.69 ± 11.88 g) were stressed by 1-min air exposure, using nets designed to fit inside the tanks and to be lifted to perform the stressful event. The experimental groups were established, in triplicate, as follows: Control – undisturbed fish (the net was also placed in the tanks but not lifted) – (NET_CTRL_), fish air-exposed twice a week (NET_2x_) and fish air-exposed four-times a week (NET_4x_). In the HYP trial, fish (IBW = 397.99 ± 16.56 g) were subjected to low levels of saturated oxygen, by injection of nitrogen in the water, for 48 h, according to the following experimental groups (in triplicate): Control – 100% saturated oxygen – (HYP_CTRL_), 30% saturated oxygen (HYP_30_) and 15% saturated oxygen (HYP_15_). Different trial times are due to differences in the nature and severity of the stressor, to which rearing protocols had to be adjusted accordingly.

### Sampling procedure

Prior to the sampling day, fish were starved for 48 h to clean the digestive tract. Nine random fish per tank were lethally anaesthetized with tricaine methanesulfonate (MS-222; Sigma Aldrich, St. Louis, Missouri, USA) for the following sampling procedures: 3 fish for *rigor mortis* index assessment, 3 fish for muscle pH measurement and 6 fish for blood collection. Blood samples of approximately 2 ml were collected from the caudal vein with a heparinized syringe and immediately centrifuged at 2000 g for 20 min. Plasma samples were immediately frozen at − 80 °C until posterior analyses. Fish for the measurement of *post-mortem* biochemical changes (pH and *rigor mortis*) were stored in polystyrene boxes with ice during the sampling period (72 h). All fish were weighed and measured.

### Plasma stress indicators’ measurement

Plasma cortisol levels were quantified using a commercial Cortisol ELISA kit RE52061 (IBL International, Hamburg, Germany), following the manufacturer’s instructions. Measurements were registered at 450 and 620 nm along with a prepared standard curve on a microplate reader Biotek Synergy 4 Hybrid Technology™ (Biotek Instruments Inc., Winooski, USA). Plasma glucose and lactate levels were assessed through commercial colorimetric kits (Spinreact, Girona, Spain), following the manufacturer’s instructions.

### Biochemical and quality characterization of fish muscle

Muscle pH measurements were performed (*n* = 3 per tank), using a waterproof pH spear for food testing (Oakton® Instruments, Nijkerk, Netherlands), in the dorsal muscle, at 0, 1, 2, 4, 6, 8, 24, 48 and 72 h after death (HAD), approximately 1–2 cm apart. At the same *post-mortem* periods, *rigor mortis* was assessed (*n* = 3 per tank) by the rigor index (RI), as previously described [85], using the formula:

$$ \mathrm{RI}\ \left(\%\right)=\left[\left({\mathrm{L}}_0-{\mathrm{L}}_{\mathrm{t}}\right)/{\mathrm{L}}_0\right]\times \kern0.37em 100. $$

L_0_ (cm) refers to the vertical distance between the base of the caudal fin and the table surface (where the anterior half of the fish is placed), measured immediately after death, whereas L_t_ (cm) corresponds to the same distance, however at selected time intervals. Fish were carefully handled during the measurements to avoid any interference with the *rigor* onset.

### Plasma proteomics analysis

#### Protein labelling

Plasma samples were diluted 80x in DIGE buffer (7 M urea, 2 M thiourea, 4% CHAPS, 30 mM Tris pH 8.5) and the protein content measured with Bradford assay using the BioRad Quick Start Bradford Dye Reagent 1X (Bio-Rad Laboratories, Hercules, California, USA) and bovine serum albumin (BSA) as standard, BioRad Bovine Serum Albumin Standard Set (Bio-Rad Laboratories, Hercules, California, USA). Samples’ pH was checked with a pH-indicator paper, Sigma-P4536 (Sigma Aldrich, St. Louis, Missouri, USA) and adjusted to 8.5 using 0.1 M NaOH. DIGE minimal labelling of 50 μg of protein was carried out using the CyDye™ DIGE fluor minimal labelling kit 5 nmol (GE Healthcare, Little Chalfont, UK), with 400 pmol fluorescent amine reactive cyanine dyes freshly dissolved in anhydrous dimethylformamide (DMF), following the manufacturer’s instructions. Labelling was achieved on ice for 30 min, in the dark, and the reaction quenched with 1 mM of lysine for 10 min. For each trial, six samples per experimental condition were labelled with Cy3 and six with Cy5 to reduce the impact of label difference, while an internal standard consisting of a pool of all samples, with equal amounts, was labelled with Cy2. Samples were randomly sorted to avoid labelling bias.

#### Protein separation by 2DE

For each strip, 150 μg of protein (50 μg from each dye) were loaded along with rehydration buffer (8 M urea, 2% CHAPS, 50 mM DTT, 0.001% bromophenol blue, 0.5% Bio-lyte 3/10 ampholyte (Bio-Rad Laboratories, Hercules, California, USA) to complete 450 μl. Passive rehydration was conducted for 15 h on 24 cm Immobiline™ Drystrips (GE Healthcare, Little Chalfont, UK) with linear pH 4–7, on an IPG Box (GE Healthcare, Little Chalfont, UK). Following, isoelectric focusing (IEF) was performed in 5 steps: 500 V gradient 1 h, 500 V step-n-hold 1 h, 1000 V gradient 1 h, 8000 V gradient 3 h and 8000 V step-n-hold 5 h40 for a total of 60.000 Vhr using Ettan IPGphor at 20 °C (GE Healthcare, Little Chalfont, UK). Focused strips were reduced and alkylated with 6 ml of equilibration buffer (50 mM Tris-HCl pH 8.8, 6 M urea, 30% (v/v) glycerol and 2% SDS) with 1% (w/v) dithiothreitol (DTT) or 2.5% (w/v) iodoacetamide (IAA) respectively for 15 min each, in constant agitation. Strips were then loaded onto 12.5% Tris-HCl SDS-PAGE gels and ran in an Ettan DALT*six* Large Vertical System (GE Healthcare, Little Chalfont, UK) at 10 mA/gel for 1 h followed by 60 mA/gel until the bromophenol blue line reaches the end of the gel, using a standard Tris-Glycine-SDS running buffer.

#### Image acquisition and analysis

CyDye-labeled gels were scanned on a Typhoon™ laser scanner 9400 (GE Healthcare, Little Chalfont, UK) at 100 μm resolution, with the appropriate laser filters for the excitation and emission wavelengths of each dye (i.e., Cy2–488/520 nm; Cy3–532/580 nm; and Cy5–633/670 nm), according to the manufacturer’s recommendations. The voltages of the Photo Multiplier Tube (PMT) were adjusted to obtain a maximum image quality with minimal signal saturation and clipping. Gel images were checked for saturation during the acquisition process using the ImageQuant TL software (GE Healthcare, Little Chalfont, UK). The final images were analysed with SameSpots software (Totallab, Newcastle, UK), including background subtraction (average normalized volume ≤ 100,000 and a spot area ≤ 500), filtering, spot detection, spot matching, normalization and statistical analysis. Spot volume ratios that showed a statistically significant difference (abundance variation of at least 1.0-fold, *P* < 0.05 - one-way ANOVA on log2-transformed normalized spot volumes) were processed for further analysis. Protein spots with statistically different intensities were manually excised from preparative gels and identified by matrix-assisted laser desorption/ionization time-of-flight/time-of-flight mass spectrometry (MALDI-TOF/TOF MS).

#### Protein identification by MALDI-TOF/TOF MS

Spots from SYPRO® Ruby-stained (InvitrogenTM, Carlsbad, CA, USA) gilthead seabream plasma 2D gels were picked and subjected to in-gel tryptic digestion, similar as reported before [86]. In this study, gel plugs were washed twice with 50 mM ammonium bicarbonate solution in 50% v/v methanol (MeOH) for 20 min and dehydrated twice for 20 min in 75% acetonitrile (ACN). Proteins were then digested with 8 μL of a solution containing 5 ng/μL trypsin (trypsin Gold, Promega) in 20 mM ammonium bicarbonate for 6 h at 37 °C. A 0.1% trifluoroacetic acid (TFA) solution in 50% ACN and a solution of 7 mg/mL α-cyano-4-hydroxycinnamic acid (CHCA) in 50% ACN/0.1% TFA were used for peptide extraction and spotting respectively. MALDI TOF/TOF analysis was performed with a TOF/TOF™ 5800 (AB SCIEX, Redwood City, CA, USA) mass spectrometer in MS and MS/MS mode. For each spot, the 10 most intense peaks of the MS spectrum were selected for MS/MS acquisition. Database interrogation was carried out over with ProteinPilot v4.5 (AB Sciex) on an in-house Mascot server version 2.6.1 (Matrix Science Ltd., London, UK).

Mass lists were searched against NCBInr database restricted to the taxonomy “other Actinopterygii” (tax ID 7898 excluding 31,033 and 7955) with the following parameters: maximum 2 missed cleavages by trypsin, peptide mass tolerance ±100 ppm, fragment mass tolerance set to 0.5 Da, carbamidomethylation of cysteine selected as fixed modification and tryptophan dioxidation, histidine, tryptophan and methionine oxidation, and tryptophan to kynurenine as variable modifications. Protein hits not satisfying a significance threshold (*P* < 0.05 and a total ion score > 60) were further searched against vertebrate EST (expressed sequence tags) database also restricted to the taxonomy “other Actinopterygii”.

#### Protein-protein interaction (PPI) network and gene ontology (GO) enrichment analyses

The theoretical molecular masses and isoelectric points (pI) of the MS identified proteins were calculated using the amino-acid sequences (in one-letter code) on the ProtParam Tool (http://us.expasy.org/tools/protparam.html). A significance cutoff was applied for the identified proteins at log-fold change ±1.0. Following, the identified proteins were blasted against *Danio rerio,* on the UniprotKB database, using the FASTA protein sequences as queries. The orthologues were mapped using STRING web tool v11.0 (https://string-db.org/) to screen for protein-protein interactions (PPI). Gene ontology (GO) enrichment analysis and network visualization and analysis were performed on Cytoscape v3.7.1 (http://www.cytoscape.org/) with the BiNGO plug-in. Important hub proteins were screened by counting the degree of connectivity of each node in the network. Over-represented GO terms were identified, using *B. rerio* as reference, by selecting the hypergeometric test with a significance threshold of 0.05 after Benjamini & Hochberg FDR correction.

### Statistical analyses

All univariate and multivariate statistical analyses were performed using R v3.5.3 for MacOSX (https://www.r-project.org). Statistical analyses of the plasma parameters and the *post-mortem* muscle biochemical changes were performed using plasma cortisol, glucose and lactate levels, muscle pH and *rigor* index as dependent variables, and the stress treatment as factor. Statistical differences between treatments were analysed independently for each trial (OC, NET and HYP). For *rigor* index and muscle pH, data were processed separately for each sampling time. Differences in plasma and muscle parameters between treatments were assessed by a one-way analysis of variance (one-way ANOVA) on log10-transformed data, except for *rigor mortis* data, which was transformed by arcsine square root. Multiple comparisons were carried out by the post-hoc Tukey HSD test. When transformed data failed the Shapiro-Wilk normality test, the non-parametric Kruskal-Wallis on ranks was used, followed by Dunn’s test. When transformed data did not verified homoscedasticity assumption by Levene’s test, statistical significance was analysed by Welch’s ANOVA, followed by Games-Howell. A significance level of α = 0.05 was used in all tests performed. Experimental data is expressed as mean ± standard deviation (SD). Principal component analysis (PCA) and hierarchical clustering analysis of the identified proteins were performed on the log2-transformed normalized spot volumes obtained from SameSpots software, with autoscaling. Heatmap was generated by comparing Z-scores of normalized spot volumes and hierarchical clustering of samples and protein spots was performed using the Euclidean distance and the maximum cluster agglomeration method as distance metrics.

## Supplementary information

**Additional file 1. **Growth performance of gilthead seabream (*Sparus aurata*) submitted to three different chronic stressors. Values are mean ± SD (*n* = 75).

**Additional file 2. **Protein spots, with statistically different relative abundances (*P* < 0.05), identified in gilthead seabream (*Sparus aurata*) blood plasma proteome from NET and HYP trials, by MALDI-TOF/TOF MS after separation by 2D-DIGE. List is given in ascending order of spot number.

**Additional file 3.** List of the overrepresented terms in the GO Enrichment analysis of the 18 proteins showing significantly differential abundance between control and NET treatments (hypergeometric test, FDR < 0.05).

## Data Availability

The authors declare that all relevant data supporting the findings of this study are available within the article (and its additional files).

## Abbreviations

ANOVA: Analysis of variances
DIGE: Differential Gel Electrophoresis
FC: Fold-change
FDR: False discovery rate
GO: Gene ontology
HAD: Hours after death
HCA: Hierarchical clustering analysis
HYP: Hypoxia
IEF: Isoelectric focusing
MALDI-TOF/TOF: Matrix-assisted-laser-desorption-ionization time-of-flight/time-of-flight
MS: Mass spectrometry
NET: Net handling
OC: Overcrowding
PCA: Principal Component Analysis
pI: Isoelectric point
PPI: Protein-protein interaction
RI: Rigor index
SD: Standard deviation

## References

[1] Huntingford FA, Adams C, Braithwaite VAA, Kadri S, Pottinger TG, Sandoe P (2006). Current issues in fish welfare. J Fish Biol.

[2] Branson EJ. Fish welfare. Branson EJ, editor. Oxford, UK: Blackwell Publishing Ltd; 2008. 300 p.

[3] Carenzi C, Verga M (2009). Animal welfare: review of the scientific concept and definition. Ital J Anim Sci.

[4] Braithwaite VA, Ebbesson LO (2014). Pain and stress responses in farmed fish. Rev Sci Tech.

[5] Cerqueira M, Millot S, Castanheira MF, Félix AS, Silva T, Oliveira GA (2017). Cognitive appraisal of environmental stimuli induces emotion-like states in fish. Sci Rep.

[6] Maria Filipa C, Luís CE, Sandie M, Stephanie R, Marie-Laure B, Børge D (2017). Coping styles in farmed fish : consequences for aquaculture. Rev Aquac.

[7] Rose JD, Arlinghaus R, Cooke SJ, Diggles BK, Sawynok W, Stevens ED (2012). Can fish really feel pain?. Fish Fish.

[8] Conte FS (2004). Stress and the welfare of cultured fish. Appl Anim Behav Sci.

[9] Selye H (1950). Stress and the general adaptation syndrome. Br Med J.

[10] Schreck CB (2010). Stress and fish reproduction: the roles of allostasis and hormesis. Gen Comp Endocrinol.

[11] Korte SM, Olivier B, Koolhaas JM (2007). A new animal welfare concept based on allostasis. Physiol Behav.

[12] Ashley PL (2007). Fish welfare: current issues in aquaculture. Appl Anim Behav Sci.

[13] Mommsen TP, Vijayan MM, Moon TW (1999). Cortisol in teleosts: dynamics, mechanisms of action, and metabolic regulation. Rev Fish Biol Fish.

[14] Wendelaar Bonga SE (1997). The stress response in fish. Physiol Rev.

[15] Pottinger TG. The stress response in fish-mechanisms, effects and measurement. In: Branson EJ, editor. Fish welfare. Oxford, UK: Blackwell Publishing Ltd; 2008. p. 32–48.

[16] Fabbri E, Moon TW (2016). Adrenergic signaling in teleost fish liver, a challenging path. Comp Biochem Physiol Part B Biochem Mol Biol.

[17] Vijayan MM, Aluru N, Leatherland JF, Leatherland JF, Woo P (2010). Stress response and the role of cortisol. Fish diseases and disorders, Vol 2: non-infectious disorders.

[18] Milligan CL, Girard SS (1993). Lactate metabolism in rainbow trout. J Exp Biol.

[19] Wood CM, Turner JD, Graham MS (1983). Why do fish die after severe exercise?. J Fish Biol.

[20] Boonstra R (2013). Reality as the leading cause of stress: rethinking the impact of chronic stress in nature. Fox C, editor. Funct Ecol.

[21] Ellis T, Yildiz HY, López-Olmeda J, Spedicato MT, Tort L, Øverli Ø (2012). Cortisol and finfish welfare. Fish Physiol Biochem.

[22] Bonier F, Martin PR, Moore IT, Wingfield JC (2009). Do baseline glucocorticoids predict fitness?. Trends Ecol Evol.

[23] Davis KB Jr, McEntire ME. Comparison of the cortisol and glucose stress response to acute confinement and resting insulin-like growth factor-I concentrations among white bass, striped bass and sunshine bass. Aquac Am B Abstr. 2006;79.

[24] Fast MD, Hosoya S, Johnson SC, Afonso LOB (2008). Cortisol response and immune-related effects of Atlantic salmon (Salmo salar Linnaeus) subjected to short- and long-term stress. Fish Shellfish Immunol.

[25] Koakoski G, Oliveira TA, da Rosa JGS, Fagundes M, Kreutz LC, Barcellos LJG (2012). Divergent time course of cortisol response to stress in fish of different ages. Physiol Behav.

[26] Madaro A, Fernö A, Kristiansen TS, Olsen RE, Gorissen M, Flik G (2016). Effect of predictability on the stress response to chasing in Atlantic salmon (Salmo salar L.) parr. Physiol Behav.

[27] Martinez-Porchas M, Martinez-Cordova LR, Ramos-Enriquez R (2009). Cortisol and glucose: reliable indicators of fish stress?. Panam J Aquat Sci.

[28] Marco-Ramell A, de Almeida AM, Cristobal S, Rodrigues P, Roncada P, Bassols A (2016). Proteomics and the search for welfare and stress biomarkers in animal production in the one-health context. Mol BioSyst.

[29] Almeida AM, Bassols A, Bendixen E, Bhide M, Ceciliani F, Cristobal S (2014). Animal board invited review: advances in proteomics for animal and food sciences. Animal.

[30] Cordeiro OD, Silva TS, Alves RN, Costas B, Wulff T, Richard N (2012). Changes in liver proteome expression of Senegalese sole (Solea senegalensis) in response to repeated handling stress. Mar Biotechnol.

[31] Alves RN, Cordeiro O, Silva TS, Richard N, de Vareilles M, Marino G (2010). Metabolic molecular indicators of chronic stress in gilthead seabream (Sparus aurata) using comparative proteomics. Aquaculture.

[32] Brunt J, Hansen R, Jamieson DJ, Austin B (2008). Proteomic analysis of rainbow trout (Oncorhynchus mykiss, Walbaum) serum after administration of probiotics in diets. Vet Immunol Immunopathol.

[33] Sanahuja I, Ibarz A (2015). Skin mucus proteome of gilthead sea bream: a non-invasive method to screen for welfare indicators. Fish Shellfish Immunol..

[34] Metzger DCH, Hemmer-Hansen J, Schulte PM (2016). Conserved structure and expression of hsp70 paralogs in teleost fishes. Comp Biochem Physiol Part D Genomics Proteomics.

[35] Ellis T, North B, Scott AP, Bromage NR, Porter M, Gadd D (2002). The relationships between stocking density and welfare in farmed rainbow trout. J Fish Biol.

[36] Naderi M, Keyvanshokooh S, Salati AP, Ghaedi A (2017). Effects of chronic high stocking density on liver proteome of rainbow trout (Oncorhynchus mykiss). Fish Physiol Biochem.

[37] Zahedi S, Akbarzadeh A, Mehrzad J, Noori A, Harsij M (2019). Effect of stocking density on growth performance, plasma biochemistry and muscle gene expression in rainbow trout (Oncorhynchus mykiss). Aquaculture..

[38] Rotllant J, Arends RJ, Mancera JM, Flik G, Wendelaar Bonga SE, Tort L (2000). Inhibition of HPI axis response to stress in gilthead sea bream (Sparus aurata) with physiological plasma levels of cortisol. Fish Physiol Biochem.

[39] Barton BA, Ribas L, Acerete L, Tort L (2005). Effects of chronic confinement on physiological responses of juvenile gilthead sea bream, Sparus aurata L., to acute handling. Aquac Res.

[40] Millán-Cubillo AF, Martos-Sitcha JA, Ruiz-Jarabo I, Cárdenas S, Mancera JM (2016). Low stocking density negatively affects growth, metabolism and stress pathways in juvenile specimens of meagre (Argyrosomus regius, Asso 1801). Aquaculture..

[41] Houslay TM, Earley RL, Young AJ, Wilson AJ (2019). Habituation and individual variation in the endocrine stress response in the Trinidadian guppy (Poecilia reticulata). Gen Comp Endocrinol.

[42] Tort L, Montero D, Robaina L, Fernández-Palacios H, Izquierdo MS (2001). Consistency of stress response to repeated handling in the gilthead sea bream Sparus aurata Linnaeus, 1758. Aquac Res.

[43] Barton BA (2002). Stress in fishes: a diversity of responses with particular reference to changes in circulating corticosteroids. Integr Comp Biol.

[44] Castanheira MF, Conceição LEC, Millot S, Rey S, Bégout M-L, Damsgård B (2015). Coping styles in farmed fish: consequences for aquaculture. Rev Aquac.

[45] Yildiz HY (2009). Reference biochemical values for three cultured Sparid fish: Striped Sea bream, Lithognathus mormyrus; common dentex, Dentex dentex; and gilthead sea bream, *Sparus aurata*. Comp Clin Path.

[46] Fanouraki E, Mylonas CC, Papandroulakis N, Pavlidis M (2011). Species specificity in the magnitude and duration of the acute stress response in Mediterranean marine fish in culture. Gen Comp Endocrinol.

[47] Naderi M, Keyvanshokooh S, Ghaedi A, Salati AP (2018). Effect of acute crowding stress on rainbow trout (Oncorhynchus mykiss): a proteomics study. Aquaculture..

[48] Omlin T, Weber J (2010). Hypoxia stimulates lactate disposal in rainbow trout. J Exp Biol.

[49] Gesto M, Lopez-Patino MA, Hernandez J, Soengas JL, Miguez JM (2013). The response of brain serotonergic and dopaminergic systems to an acute stressor in rainbow trout: a time course study. J Exp Biol.

[50] López-Patiño MA, Hernández-Pérez J, Gesto M, Librán-Pérez M, Míguez JM, Soengas JL (2014). Short-term time course of liver metabolic response to acute handling stress in rainbow trout, Oncorhynchus mykiss. Comp Biochem Physiol Part A Mol Integr Physiol.

[51] Gesto M, Soengas JL, Miguez JM (2008). Acute and prolonged stress responses of brain monoaminergic activity and plasma cortisol levels in rainbow trout are modified by PAHs (naphthalene, b-naphthoflavone and benzo(a)pyrene) treatment. Aquat Toxicol.

[52] Polakof S, Panserat S, Soengas JL, Moon TW (2012). Glucose metabolism in fish: a review. J Comp Physiol B.

[53] Weber J-M, Choi K, Gonzalez A, Omlin T (2016). Metabolic fuel kinetics in fish: swimming, hypoxia and muscle membranes. J Exp Biol.

[54] Acerete L, Reig L, Alvarez D, Flos R, Tort L (2009). Comparison of two stunning/slaughtering methods on stress response and quality indicators of European sea bass (Dicentrarchus labrax). Aquaculture..

[55] Bahuaud D, Mørkøre T, Østbye T-K, Veiseth-Kent E, Thomassen MS, Ofstad R (2010). Muscle structure responses and lysosomal cathepsins B and L in farmed Atlantic salmon (Salmo salar L.) pre- and post-rigor fillets exposed to short and long-term crowding stress. Food Chem.

[56] Poli BM, Parisi G, Scappini F, Zampacavallo G (2005). Fish welfare and quality as affected by pre-slaughter and slaughter management. Aquac Int.

[57] Bagni M, Civitareale C, Priori A, Ballerini A, Finoia M, Brambilla G (2007). Pre-slaughter crowding stress and killing procedures affecting quality and welfare in sea bass (Dicentrarchus labrax) and sea bream (Sparus aurata). Aquaculture..

[58] Matos E, Silva TS, Wulff T, Valente LMP, Sousa V, Sampaio E (2013). Influence of supplemental maslinic acid (olive-derived triterpene) on the post-mortem muscle properties and quality traits of gilthead seabream. Aquaculture..

[59] Silva TT, Matos E, Cordeiro OD, Colen R, Wulff T, Sampaio E, et al. Dietary Tools To Modulate Glycogen Storage in Gilthead Seabream Muscle: Glycerol Supplementation. 2012;. 10.1021/jf3023244 22994592

[60] Wilkinson RJ, Paton N, Porter MJR (2008). The effects of pre-harvest stress and harvest method on the stress response, rigor onset, muscle pH and drip loss in barramundi (Lates calcarifer). Aquaculture..

[61] Matos E, Gonçalves A, Nunes ML, Dinis MT, Dias J (2010). Effect of harvesting stress and slaughter conditions on selected flesh quality criteria of gilthead seabream (Sparus aurata). Aquaculture..

[62] Ayala MD, Abdel I, Santaella M, Martínez C, Periago MJ, Gil F (2010). Muscle tissue structural changes and texture development in sea bream, Sparus aurata L., during post-mortem storage. LWT - Food Sci Technol.

[63] Delbarre-Ladrat C, Chéret R, Taylor R, Verrez-Bagnis V (2006). Trends in postmortem aging in fish: understanding of proteolysis and disorganization of the myofibrillar structure. Crit Rev Food Sci Nutr.

[64] Eslamloo K, Akhavan SR, Fallah FJ, Henry MA (2014). Variations of physiological and innate immunological responses in goldfish (Carassius auratus) subjected to recurrent acute stress. Fish Shellfish Immunol..

[65] Bayne CJ, Gerwick L (2001). The acute phase response and innate immunity of fish. Dev Comp Immunol.

[66] Cray C, Zaias J, Altman NH (2009). Acute phase response in animals: a review. Comp Med.

[67] Charlie-Silva I, Klein A, Gomes JMM, Prado EJR, Moraes AC, Eto SF (2019). Acute-phase proteins during inflammatory reaction by bacterial infection: fish-model. Sci Rep.

[68] Gabay C, Kushner I (1999). Acute-phase proteins and other systemic responses to inflammation. Epstein FH, editor. N Engl J Med.

[69] Karve TM, Cheema AK (2011). Small changes huge impact: the role of protein posttranslational modifications in cellular homeostasis and disease. J Amino Acids.

[70] Roy S, Kumar V, Kumar V, Behera BK (2016). Acute phase proteins and their potential role as an Indicator for fish health and in diagnosis of fish diseases. Protein Pept Lett.

[71] Pérez-Sánchez J, Terova G, Simó-Mirabet P, Rimoldi S, Folkedal O, Calduch-Giner JA, et al. Skin Mucus of Gilthead Sea Bream (*Sparus aurata* L.). Protein Mapping and Regulation in Chronically Stressed Fish. Front Physiol. 2017;8. 10.3389/fphys.2017.00034 PMC5288811 28210224

[72] Concha MI, Molina S, Oyarzún C, Villanueva J, Amthauer R (2003). Local expression of apolipoprotein A-I gene and a possible role for HDL in primary defence in the carp skin. Fish Shellfish Immunol..

[73] Piñeiro M, Piñeiro C, Carpintero R, Morales J, Campbell FM, Eckersall PD (2007). Characterisation of the pig acute phase protein response to road transport. Vet J.

[74] Veiseth-Kent E, Grove H, Færgestad EM, Fjæra SO (2010). Changes in muscle and blood plasma proteomes of Atlantic salmon (Salmo salar) induced by crowding. Aquaculture..

[75] Magnadóttir B, Lange S. Is Apolipoprotein A-I A regulating protein for the complement system of cod (Gadus morhua L.)? Fish Shellfish Immunol. 2004;16(2):265–269. 10.1016/S1050-4648(03)00061-5 15123329

[76] Boshra H, Li J, Sunyer JO (2006). Recent advances on the complement system of teleost fish. Fish Shellfish Immunol..

[77] Sunyer JO, Tort L, Lambris JD (1997). Structural C3 diversity in fish: characterization of five forms of C3 in the diploid fish Sparus aurata. J Immunol.

[78] Porcheron G, Garénaux A, Proulx J, Sabri M, Dozois CM (2013). Iron, copper, zinc, and manganese transport and regulation in pathogenic Enterobacteria: correlations between strains, site of infection and the relative importance of the different metal transport systems for virulence. Front Cell Infect Microbiol.

[79] Funkenstein B, Rebhan Y, Dyman A, Radaelli G (2005). α2-macroglobulin in the marine fish Sparus aurata. Comp Biochem Physiol Part A Mol Integr Physiol..

[80] Dietrich MA, Hliwa P, Adamek M, Steinhagen D, Karol H, Ciereszko A (2018). Acclimation to cold and warm temperatures is associated with differential expression of male carp blood proteins involved in acute phase and stress responses, and lipid metabolism. Fish Shellfish Immunol..

[81] Kinoshita S, Itoi S, Watabe S (2001). cDNA cloning and characterization of the warm-temperature-acclimation-associated protein Wap65 from carp, *Cyprinus carpio*. Fish Physiol Biochem.

[82] Diaz-Rosales P, Pereiro P, Figueras A, Novoa B, Dios S (2014). The warm temperature acclimation protein (Wap65) has an important role in the inflammatory response of turbot (Scophthalmus maximus). Fish Shellfish Immunol..

[83] Rebl A, Goldammer T (2018). Under control: the innate immunity of fish from the inhibitors’ perspective. Fish Shellfish Immunol..

[84] Li C, Gao C, Fu Q, Su B, Chen J (2017). Identification and expression analysis of fetuin B (FETUB) in turbot (Scophthalmus maximus L.) mucosal barriers following bacterial challenge. Fish Shellfish Immunol.

[85] Erikson, U. Rigor measurements. In: S.C. Kestin, P. D. Warriss, eds. Farmed fish quality. Oxford, UK: Blackwell Science; 2001. p. 283–297.

[86] Schiener M, Hilger C, Eberlein B, Pascal M, Kuehn A, Revets D (2018). The high molecular weight dipeptidyl peptidase IV pol d 3 is a major allergen of Polistes dominula venom. Sci Rep.

