# Supplementary material for: Protein changes as robust signatures of fish chronic stress: a proteomics approach to fish welfare research
Source: BMC Genomics. 2020 Apr 19;21:309. doi: 10.1186/s12864-020-6728-4 (PMC7168993; doi:10.1186/s12864-020-6728-4)
Supplement: Supplementary file 1 — Additional file 1. Growth performance of gilthead seabream (Sparus aurata) submitted to three different chronic stressors. Values are mean ± SD (n = 75). [file 12864_2020_6728_MOESM1_ESM.pdf]

Additional file 1. Growth performance of gilthead seabream (*Sparus aurata*) submitted to three different chronic stressors. Values are mean ± SD (n = 75).

Trials/Treatments	IBW (g)[1]	FBW (g)[2]	SGR (% d^{-1})[3]
CTRL	368.63 ± 13.71	372.02 ± 48.79	0.02
OC30	371.27 ± 4.75	372.58 ± 61.45	0.01
OC45	379.15 ± 8.37	379.16 ± 71.20	0.00
CTRL	376.56 ± 12.05	345.93 ± 47.53	-0.16
NET2	374.53 ± 27.09	348.52 ± 66.42	-0.13
NET4	376.47 ± 5.86	344.04 ± 44.62	-0.17
CTRL	417.79 ± 22.73	384.71 ± 86.58	-0.15
HYP30	389.81 ± 5.73	375.40 ± 55.08	-0.07
HYP15	393.69 ± 47.54	379.16 ± 71.20	-0.07

[1]IBW - Initial body weight

[2]FBW - Final body weight

[3]SGR - Specific growth rate

[4]HSI - Hepatosomatic index
