# Supplementary material for: Protein changes as robust signatures of fish chronic stress: a proteomics approach to fish welfare research
Source: BMC Genomics. 2020 Apr 19;21:309. doi: 10.1186/s12864-020-6728-4 (PMC7168993; doi:10.1186/s12864-020-6728-4)
Supplement: Supplementary file 2 — Additional file 2. Protein spots, with statistically different relative abundances (P < 0.05), identified in gilthead seabream (Sparus aurata) blood plasma proteome from NET and HYP trials, by MALDI-TOF/TOF MS after separation by 2D-DIGE. List is given in ascending order of spot number. [file 12864_2020_6728_MOESM2_ESM.pdf]

Additional file 2. Protein spots, with statistically different relative abundances (*P* < 0.05), identified in gilthead seabream (*Sparus aurata*) blood plasma proteome from NET and HYP trials, by MALDI-TOF/TOF MS after separation by 2D-DIGE. List is given in ascending order of spot number.

Spot no[a]	Accession no.[b]	Protein ID[c]	Protein Score[d]	Total Ion Score[e]	Mw (kDa)[f]		pI[g]		PC[h]	*p-value*[i]	*q-value*[j]		FC[k]	
					T	O	T	O			T1	T2	T1	T2
Differential protein spots identified in the NET trial														
52	AEA41139.1	transferrin [Sparus aurata]	212	181	74.28	113.97	5.93	5.9	12	1.40E-02	6.76E-01	**1.35E-02**	0.32	0.85
144	AKA66306.1	complement factor B-like protein, partial [Siniperca chuatsi]	308	274	77.13	95.81	5.57	5	5	1.11E-02	1.83E-01	**8.34E-03**	**1.06**	**1.78**
146	XP_008277007.1	PREDICTED: complement factor B-like [Stegastes partitus]	327	306	84.98	93.56	5.61	5	10	3.84E-03	9.72E-02	**2.81E-03**	**1.59**	**2.38**
152	XP_008277007.1	PREDICTED: complement factor B-like [Stegastes partitus]	331	317	84.98	93.56	5.61	5	8	3.49E-04	**2.88E-02**	**2.39E-04**	**1.72**	**2.54**
202	XP_019111370.1	PREDICTED: inter-alpha-trypsin inhibitor heavy chain H3-like [Larimichthys crocea]	309	286	102.46	89.21	5.44	4.5	13	1.64E-04	3.36E-01	**1.58E-04**	-0.36	**-1.61**
	XP_010753395.2	PREDICTED: antithrombin-III [Larimichthys crocea]	197	153	51.04		5.21		13					
206	XP_019111370.1	PREDICTED: inter-alpha-trypsin inhibitor heavy chain H3-like [Larimichthys crocea]	353	331	102.46	89.21	5.44	4.5	11	2.66E-03	5.71E-01	**2.63E-03**	-0.26	**-1.66**
209	XP_019111370.1	PREDICTED: inter-alpha-trypsin inhibitor heavy chain H3-like [Larimichthys crocea]	334	295	102.46	89.21	5.44	4.6	9	2.45E-04	2.14E-01	**2.01E-04**	-0.49	**-1.76**
210	KKF19329.1	Inter-alpha-trypsin inhibitor heavy chain H3, partial [Larimichthys crocea]	363	341	87.84	89.21	5.31	4.6	14	6.21E-03	2.61E-01	**4.66E-03**	-0.39	-0.94
224	XP_017260893.1	alpha-2-macroglobulin, partial [Kryptolebias marmoratus]	91	91	40.18	85.07	5.21	5.4	1	7.01E-05	**4.10E-02**	**4.74E-05**	-0.62	**-1.74**
229	XP_017260893.1	alpha-2-macroglobulin, partial [Kryptolebias marmoratus]	115	102	40.18	85.07	5.21	5.4	1	1.78E-04	9.29E-02	**1.28E-04**	-0.46	**-1.40**
301	AWP20152.1	putative apolipoprotein B-100-like isoform 2 [Scophthalmus maximus]	186	303	422.92	75.52	4.99	5.1	27	2.45E-03	**1.71E-02**	**2.56E-03**	-0.65	-0.90
316	AWP20152.1	putative apolipoprotein B-100-like isoform 2 [Scophthalmus maximus]	219	219	422.92	75.52	4.99	5.2	25	1.39E-02	1.23E-01	**1.13E-02**	-0.88	**-1.46**

326	XP_019111370.1	PREDICTED: inter-alpha-trypsin inhibitor heavy chain H3-like [Larimichthys crocea]	129	116	102.46	77.34	5.44	4.7	10	1.11E-02	8.39E-01	**3.99E-02**	-0.10	0.50
	XP_023284462.1	antihemorrhagic factor cHLP-B-like [Seriola lalandi dorsalis]	350	367	56.45		5.97		10					
327	AEA41139.1	transferrin [Sparus aurata]	738	645	74.28	75.52	5.93	5.7	24	7.85E-03	1.00E-01	**6.12E-03**	-0.17	-0.29
328	XP_020489366.1	fetuin-B-like [Labrus bergylta]	276	246	55.36	77.34	6.04	4.5	6	2.11E-02	9.54E-01	**2.88E-02**	0.11	0.55
329	XP_020506421.1	hemopexin-like [Labrus bergylta]	194	185	49.06	77.34	5.48	4.8	6	2.35E-02	4.15E-01	**1.86E-02**	0.34	0.88
332	XP_023285742.1	alpha-1-antitrypsin homolog [Seriola lalandi dorsalis]	216	178	46.70	77.34	5.61	4.6	6	2.19E-03	1.00E+00	**5.01E-03**	0.06	0.78
355	XP_023285742.1	alpha-1-antitrypsin homolog [Seriola lalandi dorsalis]	294	259	46.70	72.01	5.61	5.3	6	4.81E-02	4.53E-01	**3.88E-02**	-0.25	-0.52
367	XP_023285742.1	alpha-1-antitrypsin homolog [Seriola lalandi dorsalis]	92	66	46.70	72.01	5.61	5.4	8	2.38E-04	2.70E-01	**2.08E-04**	**1.17**	**2.78**
385	XP_023285742.1	alpha-1-antitrypsin homolog [Seriola lalandi dorsalis]	137	96	46.70	70.32	5.61	5.1	11	7.17E-03	1.56E-01	**5.30E-03**	-0.42	-0.73
396	XP_023285742.1	alpha-1-antitrypsin homolog [Seriola lalandi dorsalis]	247	181	46.70	70.32	5.61	5.2	10	1.55E-03	6.70E-02	**1.10E-03**	-0.53	-0.91
411	XP_023285742.1	alpha-1-antitrypsin homolog [Seriola lalandi dorsalis]	325	263	46.70	70.32	5.61	5.3	12	2.13E-03	6.77E-02	**1.54E-03**	-0.43	-0.73
425	AEA41139.1	transferrin [Sparus aurata]	741	675	74.28	67.05	5.93	5.6	18	1.49E-02	3.95E-01	**1.18E-02**	0.10	0.27
427	AEA41139.1	transferrin [Sparus aurata]	544	491	74.28	67.05	5.93	5.7	15	2.47E-03	1.76E-01	**1.79E-03**	0.25	0.58
429	AEA41139.1	transferrin [Sparus aurata]	297	243	74.28	67.05	5.93	6.1	9	3.54E-02	5.16E-01	**2.92E-02**	0.14	0.39
438	XP_004544273.1	serine protease inhibitor A3K [Maylandia zebra]	108	97	45.19	65.47	6.01	4.6	5	4.19E-02	3.44E-01	**3.33E-02**	-0.33	-0.71
446	AFO64912.1	alpha-1-antitrypsin [Oplegnathus fasciatus]	562	502	46.25	65.47	5.69	4.5	8	2.72E-03	4.61E-01	**2.43E-03**	-0.19	-0.77
451	XP_006787579.1	PREDICTED: beta-2-glycoprotein 1-like [Neolamprologus brichardi]	149	114	40.18	67.05	8.70	5.9	6	1.96E-02	4.05E-01	**1.55E-02**	0.19	0.50
491	AEA41139.1	transferrin [Sparus aurata]	297	242	74.28	62.43	5.93	6	9	3.75E-02	6.96E-01	**3.44E-02**	0.13	0.43
497	AEA41139.1	transferrin [Sparus aurata]	703	626	74.28	62.43	5.93	5.9	18	5.99E-03	5.43E-02	**5.16E-03**	0.43	0.62
499	AEA41139.1	transferrin [Sparus aurata]	265	235	74.28	60.96	5.93	5.5	15	3.94E-03	5.17E-01	**3.61E-03**	0.17	0.60
502	O42175.1	RecName: Full=Apolipoprotein A-I; Short=Apo-AI; Short=ApoA-I; AltName: Full=Apolipoprotein A1; Contains: RecName: Full=Proapolipoprotein A-I; Short=ProapoA-I; Flags: Precursor	115	67	29.63	59.53	5.20	4.8	8	1.17E-03	4.30E-01	**1.37E-02**	-0.28	**1.00**
521	KKF22678.1	Fibrinogen alpha chain [Larimichthys crocea]	206	195	74.19	62.43	5.29	5.9	5	1.58E-02	9.80E-01	**2.42E-02**	0.20	**1.52**
	XP_023279907.1	fibrinogen alpha chain isoform X2 [Seriola lalandi dorsalis]	172	172	78.02		5.93		3					

544	KKF22678.1	Fibrinogen alpha chain [Larimichthys crocea]	146	133	74.19	58.13	5.29	5.5	4	7.82E-03	3.67E-01	**6.18E-03**	**1.33**	**2.26**
556	XP_018550494.1	PREDICTED: leucine-rich alpha-2-glycoprotein-like [Lates calcarifer]	274	256	37.60	58.13	5.89	4.3	4	2.70E-03	3.06E-01	**2.13E-03**	0.51	**1.20**
557	XP_023812997.1	nucleoside diphosphate kinase B [Oryzias latipes]	327	301	16.98	58.13	6.89	4.4	7	1.43E-03	3.94E-01	**1.26E-03**	0.22	0.68
558	AEA41139.1	transferrin [Sparus aurata]	124	103	74.28	56.76	5.93	5.6	11	2.34E-03	2.60E-01	**1.79E-03**	-0.46	**-2.18**
559	XP_018550494.1	PREDICTED: leucine-rich alpha-2-glycoprotein-like [Lates calcarifer]	457	415	37.60	58.13	5.89	4.4	11	1.18E-03	5.24E-01	**1.19E-03**	0.13	0.53
	AFO64912.1	alpha-1-antitrypsin [Oplegnathus fasciatus]	72	121	46.25		5.69		6					
586	AFO64912.1	alpha-1-antitrypsin [Oplegnathus fasciatus]	260	243	46.25	54.12	5.69	4.4	5	7.00E-03	**1.53E-02**	**1.30E-02**	0.48	0.46
	XP_018550494.1	PREDICTED: leucine-rich alpha-2-glycoprotein-like [Lates calcarifer]	205	184	37.60		5.89		5					
589	AEA41139.1	transferrin [Sparus aurata]	669	600	74.28	56.76	5.93	5.8	21	5.49E-03	2.82E-01	**4.17E-03**	-0.25	-0.82
591	ADM13620.1	complement component c3 [Sparus aurata]	337	326	185.32	55.43	8.08	4.9	12	1.42E-02	2.88E-01	**1.08E-02**	-0.46	**-1.22**
593	ADM13620.1	complement component c3 [Sparus aurata]	603	551	185.32	55.43	8.08	5.1	22	7.23E-03	4.42E-01	**6.02E-03**	**1.00**	**2.13**
595	ADM13620.1	complement component c3 [Sparus aurata]	667	606	185.32	55.43	8.08	5.2	31	2.79E-03	1.93E-01	**2.04E-03**	**1.08**	**2.06**
600	XP_020506421.1	hemopexin-like [Labrus bergylta]	222	253	49.06	54.12	5.48	4.4	6	1.47E-04	**4.61E-03**	**1.19E-04**	0.53	0.81
604	CCA29190.1	warm temperature acclimation protein 65-2 [Lateolabrax japonicus]	147	118	48.73	55.43	5.22	4.3	13	1.95E-04	**1.21E-02**	**1.39E-04**	0.51	0.84
	AFO64912.1	alpha-1-antitrypsin [Oplegnathus fasciatus]	93	93	46.25		5.69		2					
613	AWP20152.1	putative apolipoprotein B-100-like isoform 2 [Scophthalmus maximus]	173	175	422.92	54.12	4.99	5.4	6	1.73E-02	2.06E-01	**1.33E-02**	-0.30	-0.59
621	XP_023285742.1	alpha-1-antitrypsin homolog [Seriola lalandi dorsalis]	134	84	46.70	52.85	5.61	4.6	10	2.79E-02	1.52E-01	**2.42E-02**	-0.46	-0.89
624	AEA41139.1	transferrin [Sparus aurata]	447	391	74.28	54.12	5.93	5.8	6	3.08E-02	5.99E-01	**2.67E-02**	0.10	0.34
632	ABP35641.1	beta-actin, partial [Gobiocypris rarus]	337	286	38.97	52.85	5.20	5.2	7	6.60E-04	**2.11E-02**	**4.93E-04**	0.98	**1.65**
654	AEA41139.1	transferrin [Sparus aurata]	552	473	74.28	52.85	5.93	5.9	17	4.05E-02	3.03E-01	**3.23E-02**	0.19	0.39
663	ARI46218.1	haptoglobin [Sparus aurata]	139	103	34.94	50.40	7.62	4.9	5	3.06E-04	**2.51E-02**	**2.10E-04**	0.65	**1.09**
677	XP_019953039.1	PREDICTED: apolipoprotein B-100-like [Paralichthys olivaceus]	93	61	417.56	50.40	5.09	5	22	5.09E-06	**1.57E-03**	**3.31E-06**	**1.42**	**2.32**
	XP_026202291.1	kininogen-1-like isoform X2 [Anabas testudineus]	82	70	48.45		5.84		3					

682	AEA41139.1	transferrin [Sparus aurata]	295	273	74.28	49.21	5.93	5.8	7	6.20E-03	1.64E-01	**4.54E-03**	-0.39	-0.70
708	AEA41139.1	transferrin [Sparus aurata]	656	606	74.28	48.05	8.31	4.7	14	1.90E-03	3.57E-01	**1.59E-03**	0.15	0.46
710	ARI46218.1	haptoglobin [Sparus aurata]	345	274	74.28	48.05	5.93	5.6	10	2.72E-05	**2.04E-04**	**4.40E-05**	**1.39**	**1.53**
715	O42175.1	RecName: Full=Apolipoprotein A-I; Short=Apo-AI; Short=ApoA-I; AltName: Full=Apolipoprotein A1; Contains: RecName: Full=Proapolipoprotein A-I; Short=ProapoA-I; Flags: Precursor	327	219	34.94	46.92	7.62	5.2	17	3.64E-04	**2.26E-02**	**2.55E-04**	**1.44**	**2.20**
736	AJW65884.1	Hyaluronic acid binding protein 2 [Sparus aurata]	261	271	69.16	44.74	5.68	4.7	11	2.18E-03	3.59E-01	**1.81E-03**	-0.33	**-1.53**
737	XP_008277007.1	PREDICTED: complement factor B-like [Stegastes partitus]	128	122	29.63	46.92	5.20	4.4	2	5.79E-07	5.17E-01	**1.04E-06**	-0.31	**-2.52**
751	AEA41139.1	transferrin [Sparus aurata]	282	256	74.28	43.69	5.61	4.9	5	6.24E-05	5.81E-01	**8.46E-05**	-0.16	**-1.25**
778	AEA41139.1	transferrin [Sparus aurata]	318	275	74.28	42.66	5.93	5	6	3.94E-03	1.26E-01	**2.85E-03**	-0.28	-0.57
796	ACN54269.1	warm temperature acclimation-related 65 kDa protein [Sparus aurata]	198	165	49.16	42.66	5.93	5.2	7	4.03E-06	**1.20E-04**	**4.00E-06**	0.85	**1.13**
833	ACN54269.1	warm temperature acclimation-related 65 kDa protein [Sparus aurata]	175	159	49.16	39.72	5.41	5.2	9	4.06E-03	7.06E-02	**3.10E-03**	**-1.01**	**-1.86**
841	O42175.1	RecName: Full=Apolipoprotein A-I; Short=Apo-AI; Short=ApoA-I; AltName: Full=Apolipoprotein A1; Contains: RecName: Full=Proapolipoprotein A-I; Short=ProapoA-I; Flags: Precursor	116	104	49.16	34.44	5.41	5.3	2	6.51E-06	5.06E-02	**5.02E-06**	-0.48	**-1.40**
843	AEA41139.1	transferrin [Sparus aurata]	350	328	74.28	35.27	5.20	4.9	11	3.24E-03	7.08E-01	**3.63E-03**	-0.21	**-1.96**
864	APO15792.1	apolipoprotein Eb [Sparus aurata]	613	511	74.28	35.27	5.93	6.2	17	1.47E-04	8.47E-01	**2.59E-04**	**1.60**	**3.68**
869	APO15792.1	apolipoprotein Eb [Sparus aurata]	1070	851	30.99	35.27	4.81	4.7	24	1.93E-03	1.33E-01	**1.37E-03**	**1.41**	**2.26**
877	BAM36361.1<br>XP_022604055.1	pentraxin [Oplegnathus fasciatus]<br>kininogen-1-like [Seriola dumerili]	183<br>114	177<br>179	25.59<br>39.99	32.84	5.72<br>5.00	5.1	4<br>3	1.65E-04	**1.14E-04**	**1.39E-02**	**-1.24**	-0.73
904	AEA41139.1	transferrin [Sparus aurata]	430	411	74.28	29.86	5.93	5.6	9	3.09E-03	1.20E-01	**2.21E-03**	-0.46	**-1.17**
905	O42175.1	RecName: Full=Apolipoprotein A-I; Short=Apo-AI; Short=ApoA-I; AltName: Full=Apolipoprotein A1; Contains: RecName: Full=Proapolipoprotein A-I;	193	137	29.63	30.57	5.20	5.7	12	1.15E-03	5.45E-02	**8.11E-04**	**-1.46**	**-2.63**

		Short=ProapoA-I; Flags: Precursor												
907	O42175.1	RecName: Full=Apolipoprotein A-I; Short=Apo-AI; Short=ApoA-I; AltName: Full=Apolipoprotein A1; Contains: RecName: Full=Proapolipoprotein A-I; Short=ProapoA-I; Flags: Precursor	356	263	29.64	31.57	5.20	5.7	16	1.27E-02	8.76E-01	**4.07E-02**	0.11	-0.57
908	O42175.1	RecName: Full=Apolipoprotein A-I; Short=Apo-AI; Short=ApoA-I; AltName: Full=Apolipoprotein A1; Contains: RecName: Full=Proapolipoprotein A-I; Short=ProapoA-I; Flags: Precursor	536	381	29.63	29.86	5.20	4.7	22	7.59E-05	**3.00E-02**	**5.04E-05**	**1.27**	**2.31**
919	O42175.1	RecName: Full=Apolipoprotein A-I; Short=Apo-AI; Short=ApoA-I; AltName: Full=Apolipoprotein A1; Contains: RecName: Full=Proapolipoprotein A-I; Short=ProapoA-I; Flags: Precursor	477	297	29.63	29.86	5.20	4.4	22	1.01E-03	1.37E-01	**7.19E-04**	-0.48	**-1.16**
928	O42175.1	RecName: Full=Apolipoprotein A-I; Short=Apo-AI; Short=ApoA-I; AltName: Full=Apolipoprotein A1; Contains: RecName: Full=Proapolipoprotein A-I; Short=ProapoA-I; Flags: Precursor	236	153	29.63	29.15	5.20	5.1	15	8.23E-04	3.85E-01	**7.44E-04**	-0.23	-0.81
932	XP_023284462.1	antihemorrhagic factor cHLP-B-like [Seriola lalandi dorsalis]	445	396	56.45	29.86	5.97	6.4	7	1.06E-02	1.57E-01	**8.02E-03**	-0.48	-0.82
937	O42175.1	RecName: Full=Apolipoprotein A-I; Short=Apo-AI; Short=ApoA-I; AltName: Full=Apolipoprotein A1; Contains: RecName: Full=Proapolipoprotein A-I; Short=ProapoA-I; Flags: Precursor	226	179	29.63	28.47	5.20	4.9	10	1.04E-04	1.53E-01	**8.34E-05**	-0.56	**-1.59**
939	O42175.1	RecName: Full=Apolipoprotein A-I; Short=Apo-AI; Short=ApoA-I; AltName: Full=Apolipoprotein A1; Contains: RecName: Full=Proapolipoprotein A-I;	489	310	29.63	28.47	5.20	5.1	23	1.31E-05	**1.39E-03**	**9.29E-06**	**-1.06**	**-1.68**

		Short=ProapoA-I; Flags: Precursor												
950	O42175.1	RecName: Full=Apolipoprotein A-I; Short=Apo-AI; Short=ApoA-I; AltName: Full=Apolipoprotein A1; Contains: RecName: Full=Proapolipoprotein A-I; Short=ProapoA-I; Flags: Precursor	176	109	29.63	27.80	5.20	5.8	14	7.67E-04	**1.42E-02**	**6.37E-04**	0.48	0.73
	XP_008297163.1	PREDICTED: hemopexin-like [Stegastes partitus]	95	95	48.51		5.39		2					
954	XP_014909301.1	PREDICTED: hemopexin-like [Poecilia latipinna]	246	205	47.86	27.80	5.33	5.5	10	3.76E-04	**3.33E-04**	**6.64E-03**	**-1.03**	-0.76
959	XP_020489366.1	fetuin-B-like [Labrus bergylta]	154	123	55.36	27.80	6.04	6.4	9	6.70E-03	**2.67E-02**	**8.05E-03**	-0.47	-0.55
	XP_018541719.1	PREDICTED: C-reactive protein-like [Lates calcarifer]	132	117	25.24		5.20		7					
967	O42175.1	RecName: Full=Apolipoprotein A-I; Short=Apo-AI; Short=ApoA-I; AltName: Full=Apolipoprotein A1; Contains: RecName: Full=Proapolipoprotein A-I; Short=ProapoA-I; Flags: Precursor	373	222	29.63	26.51	5.20	4.9	22	9.35E-04	**1.78E-02**	**7.62E-04**	**-1.57**	**-3.09**
970	O42175.1	RecName: Full=Apolipoprotein A-I; Short=Apo-AI; Short=ApoA-I; AltName: Full=Apolipoprotein A1; Contains: RecName: Full=Proapolipoprotein A-I; Short=ProapoA-I; Flags: Precursor	234	120	29.63	25.88	5.20	5.1	18	1.59E-05	8.26E-02	**1.27E-05**	-0.33	-0.98
984	XP_008297163.1	PREDICTED: hemopexin-like [Stegastes partitus]	223	164	48.51	24.68	5.39	5.4	6	3.15E-04	4.99E-01	**3.43E-04**	-0.14	-0.59
987	XP_008297163.1	PREDICTED: hemopexin-like [Stegastes partitus]	180	131	48.51	25.27	5.39	5.9	8	1.48E-02	3.26E-01	**1.13E-02**	0.19	0.46
990	APO15792.1	apolipoprotein Eb [Sparus aurata]	242	220	30.99	24.68	4.81	4.7	7	4.95E-03	8.38E-01	**1.96E-02**	0.25	**-1.50**
	XP_010742296.3	apolipoprotein A-I [Larimichthys crocea]	86	78	28.82		4.62		3					
991	AEA41139.1	transferrin [Sparus aurata]	583	406	74.28	24.68	5.93	5.4	14	1.60E-03	2.11E-01	**1.20E-03**	0.19	0.48
996	APO15792.1	apolipoprotein Eb [Sparus aurata]	593	489	30.99	24.10	4.81	4.6	18	2.91E-05	6.55E-01	**4.55E-05**	-0.10	**-3.04**
	AWO96305.1	putative apolipoprotein A-IV-like [Scophthalmus maximus]	191	169	28.81		4.62		6					
997	XP_010742296.3	apolipoprotein A-I [Larimichthys crocea]	134	103	28.82	24.10	4.62	4.5	7	1.20E-05	6.89E-02	**9.41E-06**	-0.92	**-4.04**

998	O42175.1	RecName: Full=Apolipoprotein A-I; Short=Apo-AI; Short=ApoA-I; AltName: Full=Apolipoprotein A1; Contains: RecName: Full=Proapolipoprotein A-I; Short=ProapoA-I; Flags: Precursor	278	143	29.63	23.53	5.20	5.1	18	1.97E-04	2.72E-01	**1.75E-04**	-0.29	-0.99
1004	O42175.1	RecName: Full=Apolipoprotein A-I; Short=Apo-AI; Short=ApoA-I; AltName: Full=Apolipoprotein A1; Contains: RecName: Full=Proapolipoprotein A-I; Short=ProapoA-I; Flags: Precursor	423	336	29.63	22.98	5.20	5	12	5.83E-05	1.28E-01	**4.65E-05**	-0.34	**-1.10**
1014	XP_013872926.1	PREDICTED: calcium/calmodulin-dependent protein kinase type II subunit delta-like [Austrofundulus limnaeus]	168	223	41.19	22.98	6.84	5.2	7	8.52E-05	**3.75E-03**	**6.58E-05**	-0.77	**-1.27**
1016	O42175.1	RecName: Full=Apolipoprotein A-I; Short=Apo-AI; Short=ApoA-I; AltName: Full=Apolipoprotein A1; Contains: RecName: Full=Proapolipoprotein A-I; Short=ProapoA-I; Flags: Precursor	350	265	29.63	22.44	5.20	5	13	4.96E-05	**3.91E-02**	**3.37E-05**	-0.64	**-1.83**
1021	O42175.1	RecName: Full=Apolipoprotein A-I; Short=Apo-AI; Short=ApoA-I; AltName: Full=Apolipoprotein A1; Contains: RecName: Full=Proapolipoprotein A-I; Short=ProapoA-I; Flags: Precursor	305	262	29.63	22.44	5.20	5	7	1.14E-02	9.40E-01	**3.09E-02**	0.04	**-1.70**
1048	ADM13620.1	complement component c3 [Sparus aurata]	236	188	185.32	23.53	8.08	5.1	11	3.53E-04	2.79E-01	**3.04E-04**	-0.50	**-1.84**
1071	XP_020489366.1	fetuin-B-like [Labrus bergylta]	283	253	55.36	19.00	6.04	4.8	9	1.66E-02	1.38E-01	**1.34E-02**	-0.40	-0.77
1072	XP_020489366.1	fetuin-B-like [Labrus bergylta]	323	366	55.36	18.55	6.04	6.2	7	3.32E-03	1.42E-01	**2.39E-03**	-0.47	**-1.07**
1079	AEA41139.1	transferrin [Sparus aurata]	220	209	74.28	19.00	5.93	5.8	10	1.90E-03	5.08E-02	**1.41E-03**	-0.60	**-1.19**
1083	ADM13620.1	complement component c3 [Sparus aurata]	502	558	185.32	18.55	8.08	5	19	6.44E-03	8.01E-01	**7.57E-03**	0.97	**2.28**
1096	XP_023284462.1	antihemorrhagic factor cHLP-B-like [Seriola lalandi dorsalis]	292	273	56.45	18.11	5.97	5.1	7	1.07E-02	2.08E-01	**8.02E-03**	-0.35	-0.72
1103	AEA41139.1	transferrin [Sparus aurata]	502	466	74.28	18.11	5.93	5.2	10	3.70E-02	3.33E-01	**2.92E-02**	0.25	0.49
1144	O42175.1	RecName: Full=Apolipoprotein A-I; Short=Apo-AI; Short=ApoA-	202	158	29.63	15.70	5.20	4.8	10	4.86E-03	6.20E-02	**3.90E-03**	0.58	0.86

		I; AltName: Full=Apolipoprotein A1; Contains: RecName: Full=Proapolipoprotein A-I; Short=ProapoA-I; Flags: Precursor												
1151	O42175.1	RecName: Full=Apolipoprotein A-I; Short=Apo-AI; Short=ApoA-I; AltName: Full=Apolipoprotein A1; Contains: RecName: Full=Proapolipoprotein A-I; Short=ProapoA-I; Flags: Precursor	489	408	29.63	15.70	5.20	4.9	14	2.77E-04	5.66E-02	**1.88E-04**	-0.69	**-1.64**
1157	O42175.1	RecName: Full=Apolipoprotein A-I; Short=Apo-AI; Short=ApoA-I; AltName: Full=Apolipoprotein A1; Contains: RecName: Full=Proapolipoprotein A-I; Short=ProapoA-I; Flags: Precursor	136	119	29.63	15.33	5.20	5	3	2.79E-02	1.49E-01	**2.43E-02**	0.25	0.38
	XP_017288315.2	hemopexin-like [Kryptolebias marmoratus]	169	132	50.58		5.97		5					
1178	O42175.1	RecName: Full=Apolipoprotein A-I; Short=Apo-AI; Short=ApoA-I; AltName: Full=Apolipoprotein A1; Contains: RecName: Full=Proapolipoprotein A-I; Short=ProapoA-I; Flags: Precursor	332	225	29.63	14.28	5.20	5.5	18	2.81E-03	2.58E-01	**2.14E-03**	-0.31	-0.76
1221	O42175.1	RecName: Full=Apolipoprotein A-I; Short=Apo-AI; Short=ApoA-I; AltName: Full=Apolipoprotein A1; Contains: RecName: Full=Proapolipoprotein A-I; Short=ProapoA-I; Flags: Precursor	282	190	29.63	12.09	5.20	5.5	17	1.20E-03	2.32E-01	**9.22E-04**	-0.42	**-1.14**
1238	O42175.1	RecName: Full=Apolipoprotein A-I; Short=Apo-AI; Short=ApoA-I; AltName: Full=Apolipoprotein A1; Contains: RecName: Full=Proapolipoprotein A-I; Short=ProapoA-I; Flags: Precursor	445	344	29.63	11.80	5.20	5.3	12	3.01E-04	5.16E-01	**3.35E-04**	-0.25	**-1.19**
1250	O42175.1	RecName: Full=Apolipoprotein A-I; Short=Apo-AI; Short=ApoA-I; AltName: Full=Apolipoprotein	398	315	29.63	11.25	5.20	5.2	16	2.09E-04	1.30E-01	**1.56E-04**	-0.37	**-1.00**

		A1; Contains: RecName: Full=Proapolipoprotein A-I; Short=ProapoA-I; Flags: Precursor												
Differential protein spots identified in the HYP trial														
130	BAA95137.1	myosin light chain 2 [Trachurus trachurus]	734	640	19.03	82.11	4.68	5.1	11	1.38E-02	**4.41E-02**	**1.67E-02**	-0.44	-0.37
400	ADM13620.1	complement component c3 [Sparus aurata]	326	302	185.32	47.31	8.08	4.5	22	4.82E-04	**3.30E-04**	**4.04E-02**	-0.36	-0.66

[a] Spot no. – number of the spot in the 2D gel (Fig.3), attributed by the SameSpots software

[b] Accession number – NCBI accession number

[c] Protein ID – protein identification by MALDI-TOF/TOF MS

[d] Protein Score – a non-probabilistic protein score obtained by Peptide mass fingerprinting (significance was given to > 76, $P < 0.05$) and MS/MS search.

[e] Total ion Score – sum of the individual ion scores given to the peptides matched to the protein in MS/MS (significance was given when the total ion score of a maximum of 3 peptides was > 60)

[f] Theoretical and observed molecular weight (Mw) – theoretical Mw was obtained through the bioinformatic tool ProtParam (https://web.expasy.org/protparam/)

[g] Theoretical and observed isoelectric point (pI) - theoretical pI was obtained through the bioinformatic tool ProtParam (https://web.expasy.org/protparam/)

[h] PC – peptide count matched to entry

[I] *p-value* - p-value of the one-way ANOVA for the control/treated, $P < 0.05$

[j] q-value - q-value of the post-hoc multiple comparison Tukey HSD test. Bold lettering indicates significant differences (95% confidence interval).

[k] FC - Log2(fold-change) - significant changes in protein abundance (treated/control): T1 – OC30, NET2 or HYP30, T2 – OC45, NET4 or HYP15. Bold lettering indicates significant fold-changes (> 1.0 and < -1.0).
