# Supplementary material for: Protein changes as robust signatures of fish chronic stress: a proteomics approach to fish welfare research
Source: BMC Genomics. 2020 Apr 19;21:309. doi: 10.1186/s12864-020-6728-4 (PMC7168993; doi:10.1186/s12864-020-6728-4)
Supplement: Supplementary file 3 — Additional file 3. List of the overrepresented terms in the GO Enrichment analysis of the 18 proteins showing significantly differential abundance between control and NET treatments (hypergeometric test, FDR < 0.05). [file 12864_2020_6728_MOESM3_ESM.pdf]

**Additional file 3.** List of the overrepresented terms in the GO Enrichment analysis of the 18 proteins showing significantly differential abundance between control and NET treatments (hypergeometric test, FDR < 0.05).

| GO Term | Corrected *p-value* | Proteins in test set |
|---|---|---|
| GO Biological Process | | |
| response to stimulus (GO:50896) | 1.3471E-3 | ZGC:158446\|FGA\|HPX\|C3B\|SERPINC1\|TFA |
| response to chemical stimulus (GO:42221) | 2.2140E-2 | HPX\|C3B\|SERPINC1 |
| response to bacterium (GO:9617) | 2.2140E-2 | ZGC:158446\|TFA |
| response to other organism (GO:51707) | 2.3152E-2 | ZGC:158446\|TFA |
| multi-organism process (GO:51704) | 2.3152E-2 | ZGC:158446\|TFA |
| response to biotic stimulus (GO:9607) | 2.3152E-2 | ZGC:158446\|TFA |
| complement activation (GO:6956) | 2.3152E-2 | ZGC:158446 |
| activation of plasma proteins involved in acute inflammatory response (GO:2541) | 2.3152E-2 | ZGC:158446 |
| response to wounding (GO:9611) | 2.7276E-2 | ZGC:158446\|FGA |
| humoral immune response (GO:6959) | 2.7771E-2 | ZGC:158446 |
| acute inflammatory response (GO:2526) | 3.3649E-2 | ZGC:158446 |
| activation of immune response (GO:2253) | 3.4672E-2 | ZGC:158446 |
| hyaluronan metabolic process (GO:30212) | 3.4672E-2 | ZGC:110377 |
| positive regulation of immune response (GO:50778) | 3.4672E-2 | ZGC:158446 |
| protein maturation by peptide bond cleavage (GO:51605) | 3.4672E-2 | ZGC:158446 |
| platelet activation (GO:30168) | 3.4672E-2 | FGA |
| positive regulation of immune system process (GO:2684) | 3.8056E-2 | ZGC:158446 |
| regulation of immune response (GO:50776) | 3.8899E-2 | ZGC:158446 |
| immune effector process (GO:2252) | 3.8899E-2 | ZGC:158446 |
| GO Molecular function | | |
| endopeptidase inhibitor activity (GO:4866) | 2.2083E-8 | SERPINA1\|SERPINC1\|ZGC:110377\|A2ML\|KNG1 |
| endopeptidase regulator activity (GO:61135) | 2.2083E-8 | SERPINA1\|SERPINC1\|ZGC:110377\|A2ML\|KNG1 |
| peptidase regulator activity (GO:61134) | 2.2083E-8 | SERPINA1\|SERPINC1\|ZGC:110377\|A2ML\|KNG1 |
| peptidase inhibitor activity (GO:30414) | 2.2083E-8 | SERPINA1\|SERPINC1\|ZGC:110377\|A2ML\|KNG1 |
| enzyme inhibitor activity (GO:4857) | 5.2878E-8 | SERPINA1\|SERPINC1\|ZGC:110377\|A2ML\|KNG1 |
| enzyme regulator activity (GO:30234) | 9.9472E-6 | SERPINA1\|SERPINC1\|ZGC:110377\|A2ML\|KNG1 |

| | | |
|---|---|---|
| serine-type endopeptidase inhibitor activity (GO:4867) | 2.2566E-5 | SERPINA1\|SERPINC1\|ZGC:110377 |
| protein binding, bridging (GO:30674) | 1.6737E-2 | FGA |
| ferric iron binding (GO:8199) | 3.2152E-2 | TFA |
| cysteine-type endopeptidase inhibitor activity (GO:4869) | 3.5576E-2 | KNG1 |
| GO Celular Component | | |
| extracellular region (GO:5576) | 8.4350E-7 | ZGC:158446\|FGA\|HPX\|APOEB\|A2ML\|TFA |
| extracellular space (GO:5615) | 4.7732E-3 | FGA\|A2ML |
| fibrinogen complex (GO:5577) | 5.1382E-3 | FGA |
| extracellular region part (GO:44421) | 1.0052E-2 | FGA\|A2ML |
